# Association between systemic inflammatory indicators on admission and mortality in critically ill patients with diabetic kidney disease based on the MIMIC-IV database: a cohort study

**DOI:** 10.3389/fendo.2025.1503667

**Published:** 2025-05-30

**Authors:** Guo-Yin Shen, Qian-Qian Wang, Si-Ang Lv, Zhuo-Deng Huang, Ru-Lin Zhang, Jun Wu

**Affiliations:** ^1^ Department of Laboratory Medicine, Jiading Branch of Shanghai General Hospital, Shanghai Jiao Tong University School of Medicine, Shanghai, China; ^2^ Department of Pathology, The Affiliated Hospital of Youjiang Medical University for Nationalities, Baise, China; ^3^ The Key Laboratory of Molecular Pathology in Tumors of Guangxi Higher Education Institutions, Baise, China; ^4^ Department of Laboratory Medicine, Shanghai General Hospital, Shanghai Jiao Tong University School of Medicine, Shanghai, China

**Keywords:** systemic inflammatory indicators, diabetic kidney disease, mortality, MIMIC-IV, cohort study

## Abstract

**Introduction:**

Diabetic kidney disease (DKD) is linked to immunity and inflammation. We aimed to investigate if systemic inflammatory indicators can predict mortality in DKD patients in intensive care units (ICUs) and determine potential associations between them.

**Methods:**

This study included a cohort of 840 adults with DKD in the ICU. Three systemic inflammatory indicators were evaluated by peripheral blood tests: systemic immune-inflammation index (SII), systemic inflammation response index (SIRI), neutrophil-to-lymphocyte ratio (NLR). Cox regression analysis, restricted cubic spline (RCS), and Kaplan-Meier curves were used to evaluate the associations between the inflammatory indicators and the mortality of the DKD population. Receiver operating characteristic (ROC) was employed to ascertain the predictive accuracy of varied systemic inflammatory indicators.

**Results:**

After adjusting for all covariates, Cox regression analysis showed that inflammatory indicators were all significantly positively associated with 28-day mortality (SII: HR 1.39, 95% CI, 1.16-1.67, *P*<0.001; SIRI: HR 1.36, 95% CI, 1.14-1.62, *P*=0.001; NLR: HR 1.48, 95% CI, 1.20-1.84, *P*<0.001). Compared with the lowest tertile (tertile 1), participants in the highest tertile (tertile 3) had significantly increased risk of 28-day mortality (SII: HR 2.46, 95% CI, 1.51-4.02, *P*<0.001; SIRI: HR 3.31, 95% CI, 1.87-5.84, *P*<0.001; NLR: HR 3.42, 95% CI, 1.94-6.03, *P*<0.001). Furthermore, ROC curves showed that NLR and SIRI had higher predictive values than SII (NLR_AUC_ vs. SII_AUC_: 0.681 vs. 0.633, *P*=0.006; SIRI_AUC_ vs. SII_AUC_: 0.675 vs. 0.633, *P*=0.041) in predicting 28-day mortality.

**Conclusions:**

Our study demonstrated that systemic inflammatory indicators (SII, SIRI, and NLR) were positively associated with 28-day and 365-day mortality in critically ill patients with DKD. Inflammatory indicators may serve as predictors of mortality in critically ill DKD patients.

## 1 Introduction

With the increasing global prevalence of diabetes in recent decades, diabetic kidney disease (DKD) has become the most common type of chronic kidney disease (CKD) and the leading cause of end-stage renal disease (ESRD). DKD is associated with majority of the excess all-cause mortality and is a major risk factor for cardiovascular events in patients with diabetes mellitus (DM) (1). A prospective cohort study based on the National Health and Nutrition Examination Survey (NHANES) III reported that the 10-year cumulative standardized mortality increased from 7.7% among patients without DM/kidney disease to 11.5% among patients with type 2 diabetes mellitus (T2DM) but without kidney disease and to 31.1% among patients with T2DM and kidney disease. Patients who progress to ESRD have an approximately 20% annualized mortality rate (2). Furthermore, the critically ill DKD population has higher mortality and incidence of cardiovascular events due to severe infections and complications, resulting in a significant burden on the global health care expenditure (3–6). Early identification of patients at high risk of a poor prognosis is of paramount importance for the prevention of complications and reduction of both short- and long-term mortality.

Previous studies have identified hemodynamics, glycolipid metabolism disorders, and inflammation to play important roles in the progression of DKD (7–9). Among these, inflammation has drawn great interest as a modifiable risk factor that may offer preventative options. Numerous studies have shown that elevated levels of inflammatory indicators, such as tumor necrosis factor receptors (TNFRs), intercellular adhesion molecule-1 (ICAM-1), monocyte chemotactic protein-1 (MCP-1), and interleukins, are crucial in the progression of DKD. However, these are not routinely measured due to the relatively high costs and technical difficulties in clinical application (10). Of note, several composite inflammatory indicators, including but not limited to systemic immune-inflammation index (SII), systemic inflammation response index (SIRI), and neutrophil-to-lymphocyte ratio (NLR), have been demonstrated to be associated with chronic metabolic comorbidities (11, 12), and serve as prognostic predictors for malignant tumors, cardiovascular diseases, and inflammatory conditions (13–15). These systemic inflammatory indicators can simply be acquired through routine blood tests.

Although SII, SIRI, and NLR have been shown to be closely related to the progression and prognosis of a variety of diseases (16–18), the impact of these inflammatory indicators on predicting both short and long-term prognosis in critically ill patients with DKD has not been reported. Thus, we performed this study to evaluate the association between various systemic inflammatory indicators and short- and long-term mortality among DKD patients admitted to the intensive care unit (ICU) in U.S. hospitals, based on the Medical Information Mart for Intensive Care IV database.

## 2 Methods

### 2.1 Data sources and setting

A population-based cohort study was conducted using critical care databases in Medical Information Mart for Intensive Care (MIMIC-IV, version 2.2, http://mimic.mit.edu), which was built upon the MIMIC-III database (19). MIMIC-IV included >50000 patients admitted to the ICU at the Beth Israel Deaconess Medical Center (Boston, MA, USA) from 2008 to 2019. The Institutional Review Board at the Beth Israel Deaconess Medical Center granted a waiver of informed consent and approved the sharing of the research resource. The author obtained approval to access this database (certification number 53012208).

### 2.2 Study population

**Figure 1:**
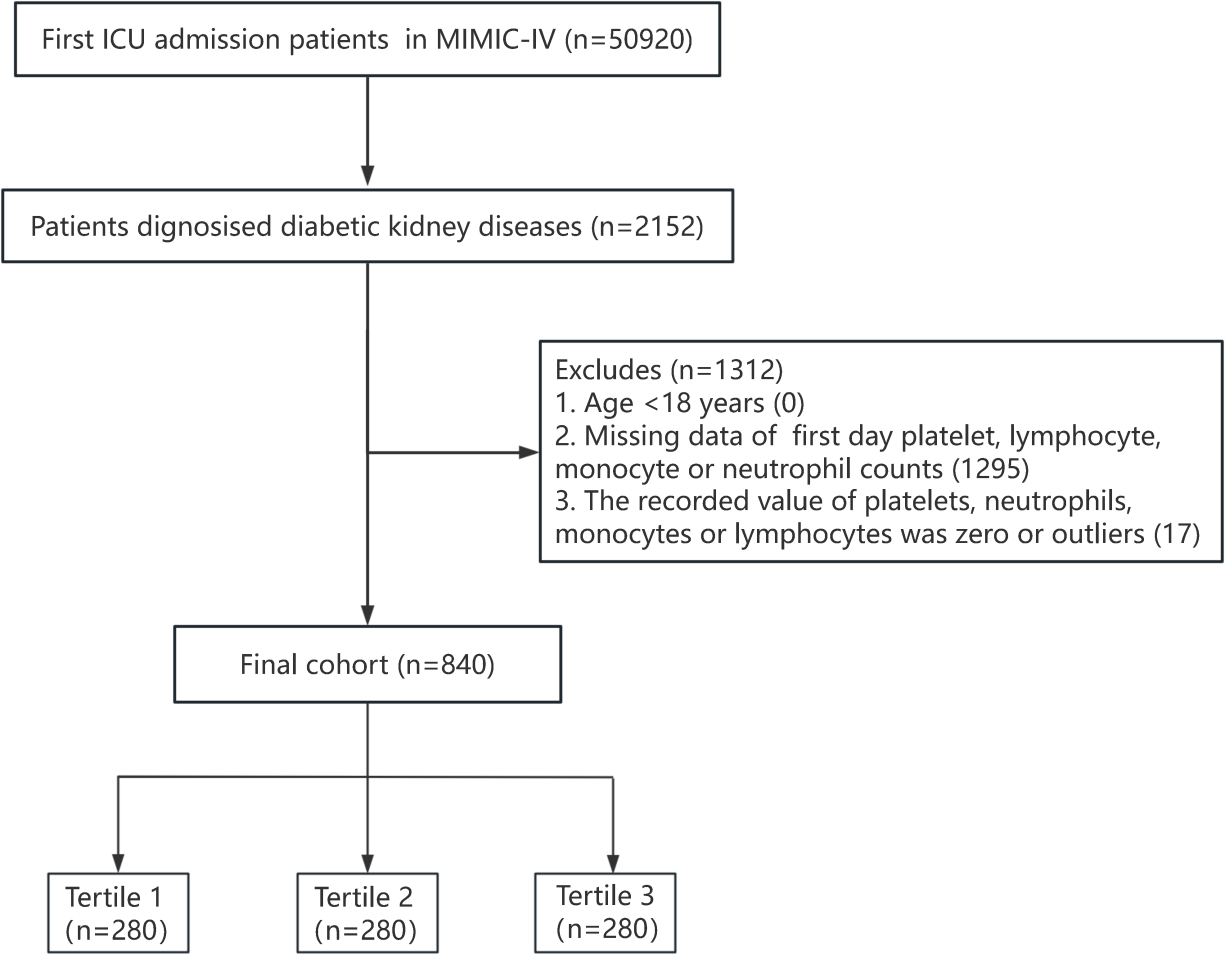
The selection process of participants in this study.

A total of 50920 adult patients admitted to the ICU for the first time were recorded in the MIMIC-IV database. Patients diagnosed as DKD according to the International Classification of Diseases ninth revision (ICD-9 codes: 24940, 24941, 25040, 25042) and tenth revision (ICD-10: E082, E0821, E0822, E0829; E102, E1021, E1022, E1029; E112, E1121, E1122, E1129; E132, E1321, E1322, E1329) were included in this study. Exclusion criteria were as follows (1): age <18 years old (2); ICU stay for <24 hours (3); missing platelet, neutrophil, monocyte, or lymphocyte counts within 24 hours of admission; and (4) the recorded value of platelets, neutrophils, monocytes, or lymphocytes was zero or outliers. A total of 840 participants were finally included. The flowchart of this study is presented in **Figure 1**.

### 2.3 Exposure variable

The systemic inflammatory indicators were calculated from absolute peripheral platelet counts (P, ×10^9^/L), neutrophil counts (N, ×10^9^/L), lymphocyte counts (L, ×10^9^/L), and monocyte counts (M, ×10^9^/L), using the following formulae:


$$SII=P\;x\;\frac{N}{L}$$

$$SIRI=N\;x\;\frac{M}{L}$$

$$NLR=\;\frac{N}{L}$$

### 2.4 Covariates

The data for this study were extracted from the MIMIC-IV database using Structured Query Language. The following data were collected (1): demographic information: age (years), gender (male/female), race (White/others) (2); vital signs: heart rate, respiratory rate, mean blood pressure (MBP), and percutaneous oxygen saturation (SpO_2_) (3); comorbidities: myocardial infarction, congestive heart failure (CHF), peripheral vascular disease (PVD), cerebrovascular disease (CVD), chronic pulmonary disease (CPD), malignancy, and severe liver disease (4); laboratory parameters on admission: potassium (mmol/L), sodium (mmol/L), hemoglobin (g/dL), bicarbonate (mmol/L), blood urea nitrogen (BUN) (mg/dL), creatinine (mmol/L), glucose (mg/dL), white blood cell (WBC) count (10^9^/L), and blood culture (5); scoring systems: Charlson comorbidity index and sequential organ failure assessment (SOFA); and (6) treatment: vasoactive agents, antibiotics, and renal replacement therapy (RRT).

### 2.5 Primary and secondary outcomes

The primary outcome of the study was 28-day mortality. Secondary outcome was 365-day mortality.

### 2.6 Statistical analysis

R software (version 4.2.2; R Foundation for Statistical Computing; http://www.R-project.org) and Free Statistics software (version 1.9.1; Beijing Free Clinical Medical Technology Co., Ltd.) were used for analyses. Normally distributed continuous variables were presented as mean ± standard deviation, non-normally distributed continuous variables were presented as median (interquartile range), and categorical variables were presented as total number (percentage). Chi-square test was used to compare categorical variables and the Kruskal-Wallis test was used to compare continuous variables. In all analyses, a two-sided *P*-value <0.05 was taken to indicate statistical significance. In this study, the missing data for covariates were minimal, all less than 0.3%, and the missing values were indirectly excluded from the analysis, detailed information on missing data and comparisons with non-missing data are presented in **Supplementary Tables S1** and **S2**, respectively. Multicollinearity was checked for the variables of multivariable analysis and was defined as a variance inflation factor of >5 (**Supplementary Table S3**). Schoenfeld residuals were used to test the proportional hazards assumptions (**Supplementary Figure S1**).

To examine the associations between the systemic inflammatory indicators and mortality in critically ill patients with DKD, SII, SIRI, and NLR were analyzed as continuous variables or tertile categorical variables. We introduced three different models using the univariate and multivariate Cox proportional hazards regression model, including the unadjusted model (crude model), the minimally adjusted model (model 1, adjusted for age, gender, and race), and the fully adjusted model (model 2, adjusted for all covariates, including model 1, heart rate, respiratory rate, MBP, SpO_2_, myocardial infarction, CHF, PVD, CVD, CPD, malignancy, severe liver disease, Charlson comorbidity index, SOFA, potassium, sodium, hemoglobin, bicarbonate, BUN, creatinine, glucose, WBC count, blood culture, vasoactive agent use, antibiotic treatment, and RRT). Hazard ratios (HRs) and 95% confidence intervals (CIs) were computed and a *P*-value <0.05 was considered statistically significant. SII, SIRI, and NLR values were naturally log-transformed when conducting regression analysis because of non-normal distribution. In addition, we performed restricted cubic spline (RCS) regression to explore the dose-response association between systemic inflammatory indicators and 28-day and 365-day mortality in DKD patients. Cumulative hazards across SII, SIRI, and NLR tertiles were shown using Kaplan–Meier curves to display the different survival probabilities among critically ill DKD patients. The log-rank test was used to compare the curves. A subgroup analysis was conducted to examine the impact of SII, SIRI, and NLR on various subgroups, such as gender, age, race, myocardial infarction, CHF, PVD, CVD, CPD, vasoactive agent use, and RRT, with a *P* for interaction <0.01 considered statically significant. Moreover, the receiver operating characteristic (ROC) was used to evaluate the prognostic capability of each inflammatory indicator in predicting the 28-day and 365-day mortality. The comparison of AUCs between NLR, SIRI and SII was performed using the DeLong test. In addition, to ensure the reliability of our findings, we conducted a sensitivity analysis using multiple imputation with five replications for the missing data.

## 3 Results

### 3.1 Baseline characteristics

A total of 840 critically ill DKD patients were included in the present study, composed of 532 men (63.3%) and 308 women (36.7%). Compared to the survivors, non-survivors were older, more likely to have a history of malignancy, had lower SPO_2_, but higher heart rate, respiratory rate, Charlson comorbidity index, SOFA, BUN, and glucose levels (all *P*<0.001). Additionally, survivors had lower SII [1128.1 (574.1, 2472.5) vs. 2263.9 (861.9, 4195.7), *P*<0.001], SIRI [3.5 (1.6, 8.3) vs. 9.4 (4.1, 17.3), *P*<0.001], and NLR [6.6 (3.7, 12.2) vs. 12.6 (7.1, 21.2), *P*<0.001]. Patients with elevated systemic inflammatory levels more likely to have CHF. They also had a lower sodium level, but higher heart rate, respiratory rate, WBC count, glucose level, serum creatinine, BUN, blood culture positivity, in-hospital mortality, and 28-day and 365-day mortality (all *P*<0.001). The baseline characteristics of critically ill DKD patients according to 28-day survival status are shown in **Table 1** and the comparisons among various tertiles of inflammatory indicators in the DKD population are found in **Supplementary Tables S4**-**S6**.

**Table 1:** The baseline information of the critically ill patients with DKD according to 28-day survival status.

Variables	Total (n =840)	28-d Survivors (n =708)	28-d Non-survivors (n =132)	*P*-value
Gender, n (%)				0.037
Female	308 (36.7)	249 (35.2)	59 (44.7)	
Male	532 (63.3)	459 (64.8)	73 (55.3)	
Age (years)	71.2 ± 11.6	70.5 ± 11.5	75.0 ± 11.4	< 0.001
Race, n (%)				0.130
White	483 (57.5)	415 (58.6)	68 (51.5)	
Others	357 (42.5)	293 (41.4)	64 (48.5)	
Vital signs				
Heart rate (beats/min)	81.7 ± 15.2	80.8 ± 14.3	86.4 ± 18.4	< 0.001
Respiratory rate (breaths/min)	19.2 ± 3.5	19.0 ± 3.3	20.5 ± 3.9	< 0.001
MBP (mmHg)	76.9 ± 10.7	77.0 ± 10.8	76.7 ± 10.5	0.803
SPO_2_ (%)	97.1 ± 2.1	97.2 ± 1.9	96.5 ± 3.1	< 0.001
Comorbidities, n%				
Myocardial infarction	307 (36.5)	251 (35.5)	56 (42.4)	0.127
CHF	472 (56.2)	386 (54.5)	86 (65.2)	0.024
PVD	130 (15.5)	108 (15.3)	22 (16.7)	0.680
CVD	120 (14.3)	97 (13.7)	23 (17.4)	0.262
CPD	211 (25.1)	181 (25.6)	30 (22.7)	0.490
Malignant cancer	87 (10.4)	62 (8.8)	25 (18.9)	< 0.001
Severe liver disease	24 (2.9)	15 (2.1)	9 (6.8)	0.007
Scoring systems				
Charlson comorbidity index	9.5 ± 2.1	9.3 ± 2.0	10.6 ± 2.3	< 0.001
SOFA	6.3 ± 3.3	6.0 ± 3.1	7.9 ± 3.9	< 0.001
Laboratory parameters				
Potassium (mmol/L)	4.6 ± 0.8	4.6 ± 0.8	4.6 ± 0.9	0.352
Sodium (mmol/L)	138.2 ± 5.4	138.1 ± 5.3	138.2 ± 5.9	0.880
Hemoglobin (g/dL)	9.4 ± 2.0	9.4 ± 2.0	9.4 ± 1.8	0.900
Bicarbonate (mmol/L)	21.4 ± 4.8	21.6 ± 4.5	20.3 ± 6.3	0.005
BUN (mg/dL)	36.0 (23.0, 57.0)	35.0 (22.0, 54.0)	47.5 (30.8, 71.0)	< 0.001
Creatinine (mmol/L)	1.9 (1.3, 3.5)	1.8 (1.3, 3.4)	2.3 (1.5, 4.0)	0.010
Glucose (mg/dL)	149.5 (112.0, 201.8)	145.0 (110.2, 191.0)	183.0 (128.2, 250.0)	< 0.001
WBC (10^9^/L)	11.3 (8.2, 15.8)	11.1 (8.1, 15.1)	12.4 (9.0, 17.4)	0.022
SII (10^9^/L)	1246.8 (602.7, 2670.9)	1128.1 (574.1, 2472.5)	2263.9 (861.9, 4195.7)	< 0.001
SIRI (10^9^/L)	4.1 (1.8, 10.2)	3.5 (1.6, 8.3)	9.4 (4.1, 17.3)	< 0.001
NLR	7.2 (3.9, 13.7)	6.6 (3.7, 12.2)	12.6 (7.1, 21.2)	< 0.001
Blood culture positivity (%)	57 (6.8)	42 (5.9)	15 (11.4)	0.023
Treatment, n%				
Vasoactive agent	382 (45.5)	313 (44.2)	69 (52.3)	0.088
Antibiotic	629 (74.9)	524 (74)	105 (79.5)	0.178
RRT	122 (14.5)	102 (14.4)	20 (15.2)	0.824

Data are presented as mean ± SD, medians (interquartile ranges) or numbers (percentages)

MBP, mean blood pressure; SPO_2_, percutaneous oxygen saturation; CHF, congestive heart failure, PVD, peripheral vascular disease; CVD, cerebrovascular disease; CPD, chronic pulmonary disease; SOFA, sequential organ failure assessment; BUN, blood urea nitrogen; WBC, white blood cell count; SII, systemic immune-inflammation index; SIRI, systemic inflammation response index; NLR, neutrophil-to-lymphocyte ratio; RRT, renal replacement therapy.

### 3.2 Associations between systemic inflammatory indicators and mortality of DKD patients in the ICU

Among all enrolled patients, the 28-day mortality was 15.7% (n=132). Multivariate Cox proportional hazards regression analysis showed that SII, SIRI, and NLR were positively associated with 28-day and 365-day mortality (**Table 2**, **Supplementary Table S7**). The associations were significant both in the crude and adjusted models. After adjusting for all covariates in model 2, the positive associations between inflammatory indicators and 28-day mortality remained stable (SII: HR 1.39, 95% CI, 1.16-1.67, *P*<0.001; SIRI: HR 1.36, 95% CI, 1.14-1.62, *P*=0.001; NLR: HR 1.48, 95% CI, 1.20-1.84, *P*<0.001). In a sensitivity analysis, a fully adjusted model for the inflammatory indicators indicated a stable positive relationship with 28-day mortality. Compared to the lowest tertile (tertile 1), participants in the highest tertile (tertile 3) had significantly increased risk of 28-day mortality (SII: HR 2.46, 95% CI, 1.51-4.02, *P*<0.001; SIRI: HR 3.31, 95% CI, 1.87-5.84, *P*<0.001; NLR: HR 3.42, 95% CI, 1.94-6.03, *P*<0.001). Participants in the middle tertile (tertile 2) also showed a higher 28-day mortality hazard compared to tertile 1, although this association did not meet the statistical significance. The *P* for trend of the three models were all <0.001.

**Table 2:** Cox proportional hazard models for 28-day mortality.

Variable	Crude model		Model 1		Model 2	
	HR (95%CI)	*P* value	HR (95%CI)	*P* value	HR (95%CI)	*P* value
Continuous ln-SII	1.43 (1.22-1.68)	<0.001	1.45 (1.23-1.70)	<0.001	1.39 (1.16-1.67)	<0.001
Categories (SII tertile)						
Tertile 1	1 (Ref)		1 (Ref)		1 (Ref)	
Tertile 2	1.12 (0.68-1.84)	0.652	1.18 (0.72-1.93)	0.518	1.36 (0.81-2.28)	0.248
Tertile 3	2.53 (1.65-3.89)	<0.001	2.61 (1.70-4.01)	<0.001	2.46 (1.51-4.02)	<0.001
*P* for trend		<0.001		<0.001		<0.001
Continuous ln-SIRI	1.48 (1.30-1.70)	<0.001	1.46 (1.28-1.67)	<0.001	1.36 (1.14-1.62)	0.001
Categories (SIRI tertile)						
Tertile 1	1 (Ref)		1 (Ref)		1 (Ref)	
Tertile 2	1.73 (1.02-2.93)	0.042	1.77 (1.05-3.01)	0.034	1.61 (0.92-2.81)	0.097
Tertile 3	3.74 (2.32-6.03)	<0.001	3.78 (2.34-6.10)	<0.001	3.31 (1.87-5.84)	<0.001
*P* for trend		<0.001		<0.001		<0.001
Continuous ln-NLR	1.74 (1.46-2.09)	<0.001	1.77 (1.48-2.11)	<0.001	1.48 (1.20-1.84)	<0.001
Categories (NLR tertile)						
Tertile 1	1 (Ref)		1 (Ref)		1 (Ref)	
Tertile 2	2.31 (1.33-4.03)	0.003	2.34 (1.34-4.08)	0.003	1.86 (1.04-3.33)	0.037
Tertile 3	4.65 (2.78-7.78)	<0.001	4.95 (2.95-8.29)	<0.001	3.42 (1.94-6.03)	<0.001
*P* for trend	<0.001		<0.001		<0.001	

In sensitivity analysis, inflammatory indicators were converted from a continuous variable to a categorical variable (tertiles).

Crude model: no covariates was adjusted.

Model 1: adjusted for age, gender, race.

Model 2: adjusted for age, gender, race, heart rate, respiratory rate, mean blood pressure, percutaneous oxygen saturation, myocardial infarction, congestive heart failure, peripheral vascular disease, cerebrovascular disease, chronic pulmonary disease, malignant cancer, severe liver disease, Charlson comorbidity index, sequential organ failure assessment, potassium, sodium, hemoglobin, bicarbonate, blood urea nitrogen, creatinine, glucose, white blood cell count, blood culture, vasoactive agent, antibiotic, renal replacement therapy.

The sensitivity analysis showed that after multiple imputation of the missing data and adjustment for all covariates, the results remained robust. (**Supplementary Table S8**, SII: HR 1.39, 95% CI, 1.16-1.67, *P*=0.001; SIRI: HR 1.36, 95% CI, 1.14-1.63, *P*=0.001; NLR: HR 1.48, 95% CI, 1.20-1.84, *P*<0.001).

**Figure 2:**
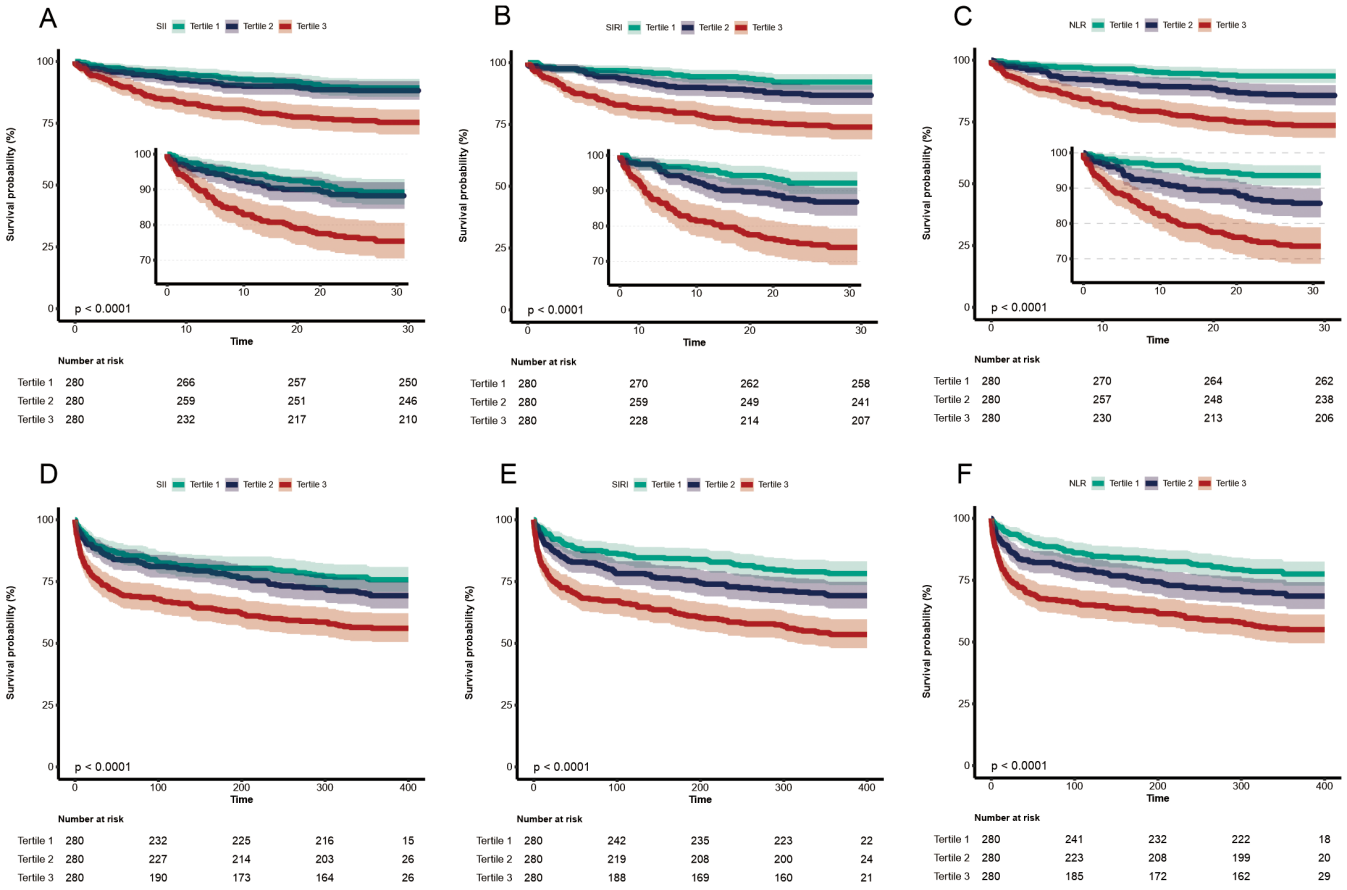
Cumulative incidence and Kaplan-Meier curve of 28-day and 365-day mortality stratified by inflammatory indicator levels. **(A–C)** 28-day mortality stratified by SII, SIRI and NLR levels, respectively; **(D–F)** 365-day mortality stratified by SII, SIRI and NLR levels, respectively. Cumulative survival rates were calculated by the Kaplan-Meier method and compared with the log-rank test. SII, systemic immune-inflammation index; SIRI, systemic inflammation response index; NLR, neutrophil-to-lymphocyte ratio.

The Kaplan-Meier curve for the SII, SIRI, and NLR tertiles is shown in **Figure 2**. The likelihood of 28-day mortality of tertile 3 was higher than tertile 1 and tertile 2 (*P*<0.001 by log-rank test). Similar trends were found in 365-day mortality.

**Figure 3:**
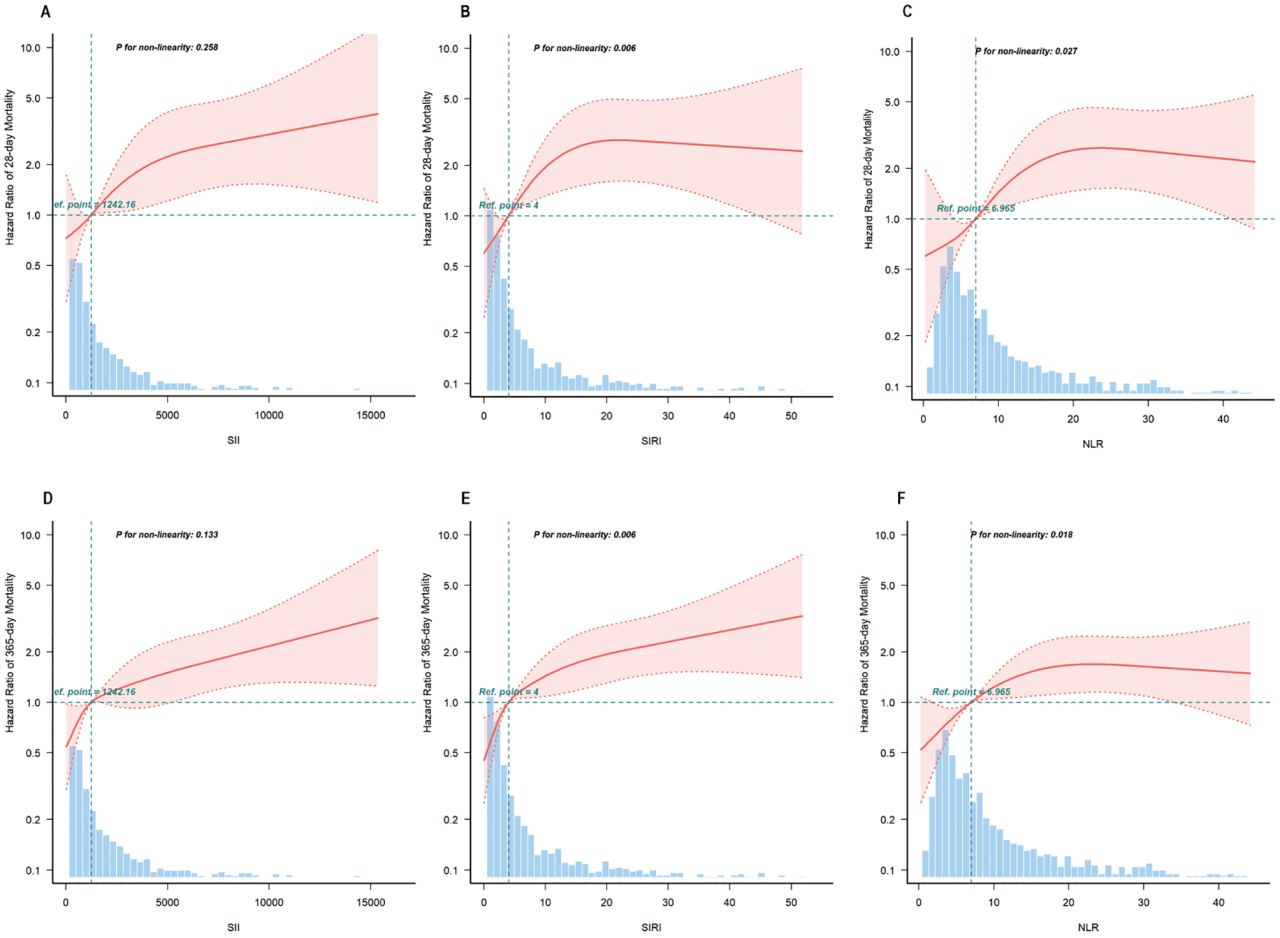
The multivariable-adjusted restricted cubic spline curves show dose-effect relationships between different systemic inflammatory indicators with the mortality of the diabetic kidney disease population in intensive care unit. **(A–C)** Restricted cubic spline for SII, SIRI and NLR on 28-day mortality risk, respectively; **(D–F)** Restricted cubic spline for SII, SIRI and NLR on 365-day mortality risk, respectively. Adjusted for age, gender, race, heart rate, respiratory rate, mean blood pressure, percutaneous oxygen saturation, myocardial infarction, congestive heart failure, peripheral vascular disease, cerebrovascular disease, chronic pulmonary disease, malignant cancer, severe liver disease, Charlson comorbidity index, sequential organ failure assessment, potassium, sodium, hemoglobin, bicarbonate, blood urea nitrogen, creatinine, glucose, white blood cell count, blood culture, vasoactive agent, antibiotic, renal replacement therapy. SII, systemic immune-inflammation index; SIRI, systemic inflammation response index; NLR, neutrophil-to-lymphocyte ratio.

RCS was used to investigate and visualize the relationships between inflammatory indicators (SII, SIRI, and NLR) and 28-day and 365-day. After adjusting for all variables, we found a linear association between SII and 28-day mortality (*P* for nonlinearity 0.258), and SII and 365-day mortality (*P* for nonlinearity 0.133). We also found a nonlinear association between SIRI and 28-day mortality (*P* for nonlinearity 0.006), SIRI and 365-day mortality *(P* for nonlinearity 0.006), NLR and 28-day mortality (*P* for nonlinearity 0.027), and NLR and 365-day mortality (*P* for nonlinearity 0.018) (**Figure 3**).

### 3.3 Subgroup analyses

Subgroup analyses were performed to assess potential effect modifications on the associations between inflammatory indicators and mortality. Regardless of age, gender, race, the presence of myocardial infarction, CHF, PVD, CVD, CPD, vasoactive agent use, and RRT, inflammatory indicators were observed to have a positive association with 28-day mortality in DKD patients, with a similar trend observed in most subgroups (**Supplementary Figure S2**-**4**). The interaction test revealed no significant differences among the subgroups, demonstrating that these factors had no significant influence on this positive relationship (*P* for interaction >0.01).

### 3.4 Predictive capacity of systemic inflammatory indicators with mortality in critically ill DKD patients

**Figure 4:**
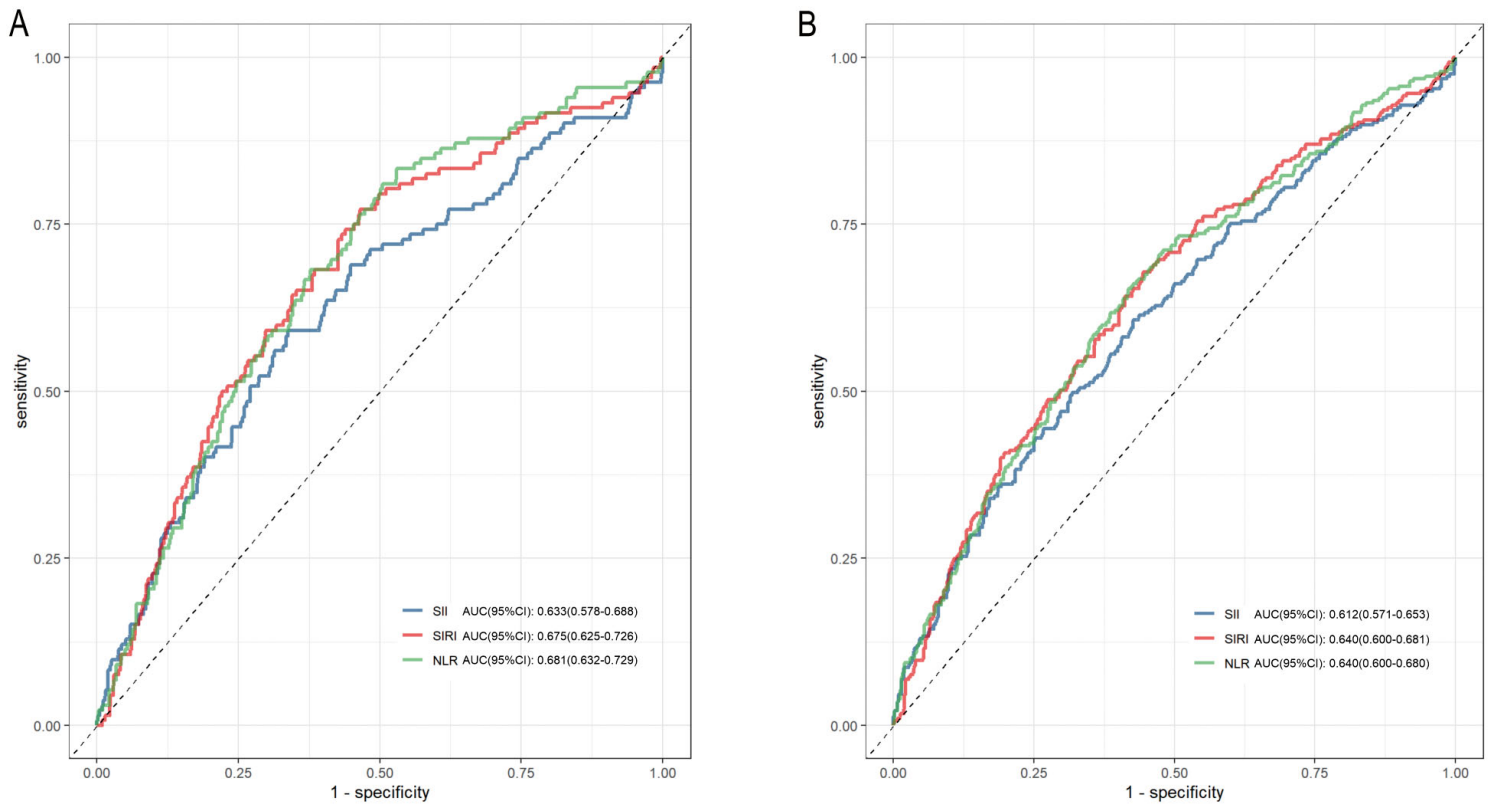
Receiver operating characteristic (ROC) curves of SII, SIRI and NLR in predicting 28-day and 365-day mortality of the diabetic kidney disease patients in intensive care unit. **(A)** ROC curve analysis of SII, SIRI, NLR and 28-day mortality; **(B)** ROC curve analysis of SII, SIRI, NLR and 365-day mortality. SII, systemic immune-inflammation index; SIRI, systemic inflammation response index; NLR, neutrophil-to-lymphocyte ratio.

ROC curves showed that NLR and SIRI had higher predictive values for 28-day mortality than SII (NLR_AUC_ vs. SII_AUC_: 0.681 vs. 0.633, *P*=0.006; SIRI_AUC_ vs. SII_AUC_: 0.675 vs. 0.633, *P*=0.041), with no significant difference between SIRI and NLR (*P*=0.785) (**Figure 4**). The area under the curve (AUC) of time-dependent ROCs showed that the inflammatory indicators had better predictive value for 28-day mortality than for 365-day mortality (**Supplementary Figure S5**). Harrell’s C-index for Cox models, net reclassification improvement (NRI), calibration plots are presented in **Supplementary Table S9**, **S10** and **Supplementary Figure S6**.

## 4 Discussion

Diabetic nephropathy is one of the common microangiopathies associated with diabetes mellitus and can significantly increase the risk of death, especially in critically ill populations. In this retrospective study, systemic inflammatory indicators (SII, SIRI, and NLR) were found to be positively correlated with short- and long-term mortality in a group of critically ill individuals with DKD from a cohort in the United States using unadjusted and adjusted Cox regression models. Sensitivity analyses showed the robustness of our findings. These indicators of inflammation (SII, SIRI, and NLR) may serve as new predictors of mortality in critically ill DKD patients. The latent progression of DKD to ESRD is significantly more prevalent than other forms of CKD, and numerous studies have shown that chronic inflammation plays a key role in the development of diabetic nephropathy (20–22). Persistent hyperglycemia in DKD patients triggers inflammatory pathways, including nuclear factor-κ B (NF-κ B), via mechanisms involving oxidative stress, the buildup of advanced glycation end products (AGEs), and activation of the renin-angiotensin system (RAS). This process triggers the release of a cascade of pro-inflammatory cytokines, leading to glomerulosclerosis, podocyte injury, and renal tubulointerstitial fibrosis. Insulin resistance and lipotoxicity (e.g., elevated free fatty acids) further stimulate macrophages and T cells. These inflammatory mediators may exert local effects within the kidneys and also propagate systemic inflammatory responses. As renal function deteriorates, the accumulation of uremic toxins amplifies inflammation while suppressing immune cell function (e.g., impaired neutrophil chemotaxis and diminished T-cell responses), thereby increasing susceptibility to infections. Concurrently, inflammatory cytokines enhance endothelial adhesion molecule expression, accelerate foam cell formation, and destabilize atherosclerotic plaques, collectively elevating the risk of cardiovascular events. In summary, systemic inflammation in DKD functions both as a pathological driver and a “bridge” connecting multisystem damage. Uremia-associated inflammation, cardiovascular risk, and infection burden are intricately intertwined, synergistically contributing to adverse clinical outcomes (18, 23, 24).

The association between inflammatory markers and nephropathy has been investigated in several recent studies across diverse populations (25, 26). Guo et al. demonstrated that a high SII was associated with the development of diabetic nephropathy in 3937 diabetic cases in the United States, based on the NHANES database (27). Results of a cross-sectional study of SII and diabetic nephropathy in a Chinese population of 1922 cases by Yan et al. were consistent with those previously reported by Guo (28). Liu et al. indicated that SII and SIRI were associated with an elevated risk of developing DKD and that SII was linked to the prognosis of DKD (10). A cross-sectional study on a Chinese population with type 2 DM showed that high levels of NLR and platelet-to-lymphocyte ratio (PLR) were predictive of DKD development and were closely related to renal function (5). Prior research has shown a positive correlation between elevated SII and SIRI and the prevalence of diabetic retinopathy (DR), as well as the independent risk factors for estimating the incidence of DR in patients with diabetes (29). A study based on a population of 6880 cases from the NHANES database confirmed that there was a significant positive correlation between systemic inflammatory markers (SII, NLR, and PLR) and all-cause mortality among patients with CKD; among these markers, NLR and lymphocyte-to-monocyte (LMR) were identified as the major predictors of survival in patients with CKD (30). Another study corroborated the findings of the previous research, demonstrating that elevated levels of SIRI were associated with an increased risk of cardiovascular and all-cause mortality in patients with CKD. The predictive power of SIRI for all-cause mortality was also evaluated, with an AUC of 0.624 (26). Nevertheless, no study has hitherto reported the relationship between levels of inflammatory markers and the short- and long-term mortality outcomes of a population with severe diabetic nephropathy. Our study precisely addresses this gap. Our findings were generally consistent with those of previous studies indicating that elevated inflammatory indicators (SII, SIRI, and NLR) were positively associated with short- and long-term mortality in the critically ill DKD population. Participants in the highest tertile exhibited a considerably elevated risk of mortality at both 28 and 365 days compared to those in the lowest tertile, even after adjusting for potential confounding variables. Of note, our findings revealed that in predicting 28-day mortality, NLR and SIRI exhibited greater efficacy than SII.

There has been increasing evidence for the role of immune-mediated inflammation and oxidative stress in the progression of DKD (31). Imbalances in several pro-inflammatory and anti-inflammatory cytokines have been reported to be associated with the development of DKD, including memory B cells, gamma delta T cells, mononuclear phagocytic cells (MNPs), neutrophils, platelets, tumor necrosis factor alpha (TNF-α), 8-Oxo-7,8-dihydro-2-deoxyguanosine(8oxodG), interleukin 6 (IL-6), and C-reactive protein (CRP) (20, 32–34). Neutrophils are important components of the innate immune system, which play a crucial role in the initiation and regulation of inflammatory processes. Neutrophil gelatinase-associated lipocalin (NGAL) released by neutrophils, has been identified as a potential predictor of DKD and its progression. Additionally, neutrophils may promote DKD progression via the secretion of neutrophil elastase (NE), a pro-inflammatory enzyme. The pro-inflammatory effect of NE has been substantiated in numerous disease models. Furthermore, NE directly induces renal cell damage, thereby accelerating the progression of DKD in DM patients (35). T cells secrete the cytokines interferon gamma (IFN-γ) and TNF-α, which facilitate the promotion of inflammation by activating macrophages and endothelial cells. B lymphocytes are implicated in the pathophysiology of renal injury through the production of immunoglobulins, the formation of immune complex deposits, and the activation of the complement system (36). Clinical studies have indicated that patients diagnosed with DKD exhibit a reduction in lymphocyte count. The underlying mechanisms responsible for the aberrant recruitment of lymphocytes and the reduction in circulating lymphocytes within the kidney remain uncertain. Further investigation is necessary to elucidate the precise mechanism. Macrophages represent a vital source of inflammatory cytokines. In DKD populations, cross-talk between the Notch pathway and NF-κ B signaling in macrophages has been found to contribute to the polarization of macrophages. The hyperpolarized macrophages secrete substantial quantities of pro-inflammatory cytokines, thereby exacerbating the inflammatory response and fibrotic process in intrinsic kidney cells. Additionally, mesangial cells have been proven to respond to high glucose-treated macrophage-derived exosomes by promoting the activation of the NLRP3 inflammasome and autophagy deficiency, thereby contributing to the development of diabetic nephropathy (37, 38). In addition to their primary physiological function in hemostasis, platelets play a crucial role in inflammatory processes. Activation of platelets is a well-documented phenomenon in patients with diverse forms of renal diseases. A substantial body of evidence from multiple studies indicates that patients with DKD exhibit enhanced platelet activation, which orchestrates a broad diversity of platelet responses, including the secretion of inflammatory factors, platelet-leukocyte interaction and profibrotic responses; collectively, these processes contribute to the deterioration of renal function (39–41). Moreover, concomitant platelet thrombosis contributes to the progression of DKD (42). Moreover, thrombocytopenia is a prevalent condition among critically ill patients. A multitude of studies have confirmed a correlation between reduced platelet count and adverse prognosis in ICU patients (43–45). Since SII is calculated by multiplying platelet counts by neutrophil counts divided by lymphocyte counts, a reduction in platelet count will result in a corresponding reduction in the value of the SII. Thus, this provides an explanation as to why NLR and SIRI demonstrate superior predictive values in comparison to SII in our study, which is contrary to the findings in other DKD populations.

Composite markers are a widely available method that offers a number of benefits, including non-invasiveness, ease of measurement, and cost-effectiveness. These markers are calculated by counting four types of circulating immune cells: neutrophils, lymphocytes, monocytes, and platelets. Composite marker levels provide a more comprehensive clinical picture than that which can be obtained from a single type of peripheral blood sample. The integration of inflammatory indicators provides a predictive value derived from the analysis of two or three types of circulating immune cells, which may reflect the equilibrium of the patient’s inflammatory, immune, and thrombotic pathways. Such techniques have gained widespread use in the fields of oncology and cardiovascular medicine. SII was demonstrated to be a valuable prognostic indicator for a range of cancers (46–48). Recent studies have shown that SII, SIRI, and NLR have prognostic value for cardio-cerebrovascular diseases. Hu et al. demonstrated that the in-hospital mortality of patients with acute ischemic stroke (AIS) predicted by the SII had an area under the ROC curve of 0.65, indicating that the SII exhibited a marginally superior discriminative capacity compared to the NLR and PLR (49–51). Conversely, our findings revealed that NLR and SIRI demonstrated a more pronounced predictive efficacy for 28-day mortality than SII in a severe DKD population.

To the best of our knowledge, no prior research has examined the relationship between the three inflammatory indicators (SII, SIRI, and NLR) and mortality in a population with severe DKD in an extensive retrospective cohort study. Our study demonstrated that the levels of the three inflammatory indicators (SII, SIRI, and NLR) were positively associated with both 28-day short-term and 365-day long-term mortality in the critically ill DKD population, and found that NLR and SIRI have better predictive values for 28-day mortality than SII. Another strength of our study is how the statistical analysis was performed. We analyzed the exposure variables (SII, SIRI and NLR) not only as continuous variables but also as categorical variables, and calculated the hazard ratios using Cox regression models. Such a method can minimize the incidence of contingency in statistical analysis and enhance the reliability of the final results. Finally, in light of their cost-effectiveness, convenient availability, and reliability, SII, SIRI, and NLR have proven to be invaluable tools for effectively monitoring patients with DKD in the ICU setting.

Our study had several limitations. Firstly, as a single-center retrospective study, selection bias was inevitable, the potential for overfitting due to limited sample size cannot be excluded. Secondly, the inflammatory indicators were calculated using only the initial test results after the patient entered the ICU, however, inflammatory markers often fluctuate during ICU stays, and mortality risk may depend on time-varying trends. Thirdly, this database did not provide critical markers of DKD progression, including albuminuria, eGFR, or DKD stage. The potential impact of these key indicators cannot be ruled out. In subsequent research, we aim to investigate the relationship between the dynamic trajectories of inflammatory markers and mortality risk, and conduct additional analyses adjusting for critical DKD progression markers, such as albuminuria, eGFR, and DKD stage, to determine whether inflammatory markers persist as independent predictors of prognosis in critically ill DKD patients.

## 5 Conclusion

Our study demonstrated that SII, SIRI, and NLR were positively associated with the risk of 28-day and 365-day mortality among DKD patients in the ICU. Additionally, the predictive values of NLR and SIRI for 28-day mortality were better than that of SII. These findings can help clinicians in dynamic monitoring of the critically ill DKD population.

## Data Availability

The original contributions presented in the study are included in the article/**Supplementary Material**. Further inquiries can be directed to the corresponding authors.
