# Supplementary material for: Association between systemic inflammatory indicators on admission and mortality in critically ill patients with diabetic kidney disease based on the MIMIC-IV database: a cohort study
Source: Front Endocrinol (Lausanne). 2025 May 30;16:1503667. doi: 10.3389/fendo.2025.1503667 (PMC12162917; doi:10.3389/fendo.2025.1503667)
Supplement: Supplementary file 1 [file Table1.docx]

Association between systemic inﬂammatory indicators on admission and mortality in critically ill patients with diabetic kidney disease based on the MIMIC-IV database: a cohort study

Supplementary Material

# Supplementary Figures and Tables

## Supplementary Tables

Table S1 The detailed information on missing data

| Variable | Missing data (n) | Missing data (%) |
| --- | --- | --- |
| Potassium (mmol/L) | 1 | 0.119 |
| Heart rate (beats/min) | 1 | 0.119 |
| MBP (mmHg) | 1 | 0.119 |
| Respiratory rate (breaths/min) | 1 | 0.119 |
| SOFA | 1 | 0.119 |
| Glucose (mg/dL) | 2 | 0.2381 |
| SPO2(%) | 2 | 0.2381 |

MBP, mean blood pressure; SOFA, sequential organ failure assessment; SPO_2_, percutaneous oxygen saturation.

Table S2 Comparison of variables between the population with missing data and the population participating in the study.

| Variables | Total (n = 840) | Missing  (n = 5) | Participating  (n = 835) | *P* |
| --- | --- | --- | --- | --- |
| Gender, n (%) | |  |  | 1.000 |
| Female | 308 (36.7) | 2 (40) | 306 (36.6) |  |
| Male | 532 (63.3) | 3 (60) | 529 (63.4) |  |
| Age (years) | 71.2 ± 11.6 | 63.9 ± 17.2 | 71.3 ± 11.6 | 0.155 |
| Race, n (%) | |  |  | 1.000 |
| White | 483 (57.5) | 3 (60) | 480 (57.5) |  |
| Others | 357 (42.5) | 2 (40) | 355 (42.5) |  |
| Heart rate (beats/min) | 81.7 ± 15.2 | 79.5 ± 10.0 | 81.7 ± 15.2 | 0.769 |
| MBP (mmHg) | 76.9 ± 10.7 | 80.8 ± 15.5 | 76.9 ± 10.7 | 0.468 |
| Respiratory rate (breaths/min) | 19.2 ± 3.5 | 19.9 ± 4.4 | 19.2 ± 3.5 | 0.703 |
| SPO_2_ (%) | 97.1 ± 2.1 | 97.7 ± 1.1 | 97.1 ± 2.1 | 0.646 |
| Myocardial infarction, n (%) | 307 (36.5) | 3 (60) | 304 (36.4) | 0.361 |
| CHF, n (%) | 472 (56.2) | 3 (60) | 469 (56.2) | 1.000 |
| PVD, n (%) | 130 (15.5) | 2 (40) | 128 (15.3) | 0.173 |
| CVD, n (%) | 120 (14.3) | 0 (0) | 120 (14.4) | 1.000 |
| CPD, n (%) | 211 (25.1) | 1 (20) | 210 (25.1) | 1.000 |
| Malignant cancer, n (%) | 87 (10.4) | 0 (0) | 87 (10.4) | 1.000 |
| Severe liver disease, n (%) | 24 (2.9) | 0 (0) | 24 (2.9) | 1.000 |
| Charlson comorbidity index | 9.5 ± 2.1 | 8.8 ± 1.6 | 9.5 ± 2.1 | 0.478 |
| SOFA | 6.3 ± 3.3 | 5.2 ± 2.2 | 6.3 ± 3.3 | 0.509 |
| Antibiotic, n (%) | 629 (74.9) | 4 (80) | 625 (74.9) | 1.000 |
| Blood culture positivity, n (%) | 57 (6.8) | 0 (0) | 57 (6.8) | 1.000 |
| Vasoactive agent, n (%) | 382 (45.5) | 2 (40) | 380 (45.5) | 1.000 |
| RRT, n (%) | 122 (14.5) | 0 (0) | 122 (14.6) | 1.000 |
| Sodium (mmol/L) | 138.2 ± 5.4 | 134.8 ± 10.8 | 138.2 ± 5.3 | 0.163 |
| Potassium (mmol/L) | 4.6 ± 0.8 | 4.4 ± 1.0 | 4.6 ± 0.8 | 0.714 |
| Bicarbonate (mmol/L) | 21.4 ± 4.8 | 18.8 ± 5.0 | 21.4 ± 4.8 | 0.231 |
| Hemoglobin (g/dL) | 9.4 ± 2.0 | 9.6 ± 2.2 | 9.4 ± 2.0 | 0.792 |
| Creatinine (mmol/L) | 1.9 (1.3, 3.5) | 1.1 (1.1, 2.1) | 1.9 (1.3, 3.5) | 0.193 |
| BUN (mg/dL) | 36.0 (23.0, 57.0) | 15.0 (15.0, 27.0) | 36.0 (24.0, 57.0) | 0.021 |
| Glucose (mg/dL) | 149.5 (112.0, 201.8) | 228.0 (198.0, 237.0) | 149.0 (112.0, 201.0) | 0.139 |
| WBC (10^9^/L) | 11.3 (8.2, 15.8) | 16.4 (13.6, 19.5) | 11.3 (8.2, 15.7) | 0.185 |
| SII (10^9^/L) | 1246.8 (602.7, 2670.9) | 802.3 (641.5, 3903.7) | 1247.0 (602.6, 2667.5) | 0.958 |
| SIRI (10^9^/L) | 4.1 (1.8, 10.2) | 2.5 (1.7, 11.9) | 4.1 (1.8, 10.2) | 0.805 |
| NLR | 7.2 (3.9, 13.7) | 6.2 (3.4, 9.4) | 7.2 (4.0, 13.7) | 0.622 |

Data are presented as mean ±SD, medians (interquartile ranges) or numbers (percentages)

MBP, mean blood pressure; SPO_2_, percutaneous oxygen saturation; CHF, congestive heart failure; PVD, peripheral vascular disease; CVD, cerebrovascular disease; CPD, chronic pulmonary disease; SOFA, sequential organ failure assessment; RRT, renal replacement therapy; BUN, blood urea nitrogen; WBC, white blood cell count; SII, systemic immune-inflammation index; SIRI, systemic inflammation response index; NLR, neutrophil-to-lymphocyte ratio.

Table S3 Diagnosis of collinearity among variables in multivariable analysis.

| Variables | VIF | | |
| --- | --- | --- | --- |
|  | SII | SIRI | NLR |
| Gender | 1.217 | 1.216 | 1.22 |
| Age | 1.488 | 1.381 | 1.421 |
| Race | 1.172 | 1.183 | 1.179 |
| Heart rate | 1.344 | 1.396 | 1.387 |
| MBP | 1.412 | 1.411 | 1.417 |
| Respiratory rate | 1.466 | 1.447 | 1.449 |
| SPO_2_ | 1.221 | 1.242 | 1.235 |
| Myocardial infarction | 1.238 | 1.216 | 1.215 |
| CHF | 1.237 | 1.25 | 1.225 |
| PVD | 1.173 | 1.172 | 1.166 |
| CVD | 1.321 | 1.27 | 1.276 |
| CPD | 1.256 | 1.248 | 1.266 |
| Malignant cancer | 2.313 | 2.267 | 2.301 |
| Severe liver disease | 1.636 | 1.604 | 1.597 |
| Charlson comorbidity index | 2.522 | 2.477 | 2.526 |
| SOFA | 2.68 | 2.542 | 2.548 |
| Antibiotic | 1.307 | 1.297 | 1.3 |
| Blood culture positivity | 1.268 | 1.22 | 1.423 |
| Vasoactive agent | 2.17 | 2.162 | 2.184 |
| RRT | 1.501 | 1.527 | 1.545 |
| Sodium | 1.332 | 1.317 | 1.321 |
| Potassium | 1.32 | 1.312 | 1.307 |
| Creatinine | 2.359 | 2.414 | 2.474 |
| BUN | 1.778 | 1.829 | 1.836 |
| Bicarbonate | 1.234 | 1.238 | 1.228 |
| Glucose | 1.191 | 1.148 | 1.147 |
| WBC | 1.283 | 1.374 | 1.264 |
| Hemoglobin | 1.3 | 1.303 | 1.262 |

VIF = 1/(1-R^2^). VIF, variance inflation factors; SII, systemic immune-inflammation index; SIRI, systemic inflammation response index; NLR, neutrophil-to-lymphocyte ratio; MBP, mean blood pressure; SPO_2_, percutaneous oxygen saturation; CHF, congestive heart failure; PVD, peripheral vascular disease; CVD, cerebrovascular disease; CPD, chronic pulmonary disease; SOFA, sequential organ failure assessment; RRT, renal replacement therapy; BUN, blood urea nitrogen; WBC, white blood cell count.

Table S4 Characteristics and outcomes of participants categorized by SII

| Variables | Total  (n = 840) | SII | | | *P* -value |
| --- | --- | --- | --- | --- | --- |
|  |  | Tertile 1  (n = 280) | Tertile 2  (n = 280) | Tertile 3  (n = 280) |  |
| Gender, n (%) | |  |  |  | 0.088 |
| Female | 308 (36.7) | 101 (36.1) | 91 (32.5) | 116 (41.4) |  |
| Male | 532 (63.3) | 179 (63.9) | 189 (67.5) | 164 (58.6) |  |
| Age (years) | 71.2 ± 11.6 | 71.7 ± 11.6 | 70.7 ± 11.4 | 71.2 ± 11.8 | 0.589 |
| Race, n (%) | |  |  |  | 0.791 |
| White | 483 (57.5) | 157 (56.1) | 161 (57.5) | 165 (58.9) |  |
| Others | 357 (42.5) | 123 (43.9) | 119 (42.5) | 115 (41.1) |  |
| *Vital signs* |  |  |  |  |  |
| Heart rate (beats/min) | 81.7 ± 15.2 | 79.9 ± 12.6 | 80.8 ± 15.2 | 84.5 ± 17.0 | < 0.001 |
| Respiratory rate (breaths/min) | 19.2 ± 3.5 | 18.4 ± 3.2 | 18.9 ± 3.2 | 20.3 ± 3.7 | < 0.001 |
| MBP (mmHg) | 76.9 ± 10.7 | 75.9 ± 11.1 | 77.5 ± 10.4 | 77.4 ± 10.6 | 0.174 |
| SPO_2_ (%) | 97.1 ± 2.1 | 97.4 ± 1.9 | 97.2 ± 2.0 | 96.7 ± 2.3 | 0.001 |
| *Comorbidities, n (%)* |  |  |  |  |  |
| Myocardial infarction | 307 (36.5) | 105 (37.5) | 102 (36.4) | 100 (35.7) | 0.907 |
| CHF | 472 (56.2) | 131 (46.8) | 159 (56.8) | 182 (65) | < 0.001 |
| PVD | 130 (15.5) | 34 (12.1) | 41 (14.6) | 55 (19.6) | 0.044 |
| CVD | 120 (14.3) | 49 (17.5) | 36 (12.9) | 35 (12.5) | 0.169 |
| CPD | 211 (25.1) | 56 (20) | 60 (21.4) | 95 (33.9) | < 0.001 |
| Malignant cancer | 87 (10.4) | 31 (11.1) | 21 (7.5) | 35 (12.5) | 0.135 |
| Severe liver disease | 24 (2.9) | 13 (4.6) | 5 (1.8) | 6 (2.1) | 0.087 |
| *Scoring systems* |  |  |  |  |  |
| Charlson comorbidity index | 9.5 ± 2.1 | 9.3 ± 2.0 | 9.3 ± 2.1 | 9.8 ± 2.2 | 0.004 |
| SOFA | 6.3 ± 3.3 | 6.7 ± 3.3 | 6.0 ± 3.2 | 6.2 ± 3.4 | 0.037 |
| *Laboratory parameters* |  |  |  |  |  |
| Potassium (mmol/L) | 4.6 ± 0.8 | 4.5 ± 0.8 | 4.6 ± 0.8 | 4.6 ± 0.9 | 0.165 |
| Sodium (mmol/L) | 138.2 ± 5.4 | 139.0 ± 4.8 | 138.7 ± 5.0 | 136.8 ± 6.0 | < 0.001 |
| Hemoglobin (g/dL) | 9.4 ± 2.0 | 9.1 ± 1.9 | 9.3 ± 2.1 | 9.8 ± 2.0 | < 0.001 |
| Bicarbonate (mmol/L) | 21.4 ± 4.8 | 21.6 ± 4.1 | 21.8 ± 4.5 | 20.7 ± 5.7 | 0.018 |
| BUN (mg/dL) | 36.0 (23.0, 57.0) | 30.0 (19.8, 45.2) | 34.5 (24.8, 61.0) | 45.0 (29.0, 66.0) | < 0.001 |
| Creatinine (mmol/L) | 1.9 (1.3, 3.5) | 1.6 (1.2, 2.8) | 1.8 (1.3, 3.3) | 2.5 (1.5, 4.1) | < 0.001 |
| Glucose (mg/dL) | 149.5 (112.0, 201.8) | 127.0 (101.2, 163.5) | 152.0 (113.8, 198.8) | 177.5 (125.8, 251.2) | < 0.001 |
| WBC (10^9^/L) | 11.3 (8.2, 15.8) | 9.0 (6.3, 11.8) | 10.9 (8.4, 14.3) | 14.9 (11.4, 19.9) | < 0.001 |
| Blood culture positivity (%) | 57 (6.8) | 9 (3.2) | 14 (5) | 34 (12.1) | < 0.001 |
| *Treatment, n (%)* |  |  |  |  |  |
| Vasoactive agent | 382 (45.5) | 148 (52.9) | 118 (42.1) | 116 (41.4) | 0.010 |
| Antibiotic | 629 (74.9) | 212 (75.7) | 187 (66.8) | 230 (82.1) | < 0.001 |
| RRT | 122 (14.5) | 32 (11.4) | 36 (12.9) | 54 (19.3) | 0.019 |
| *Death events, n (%)* |  |  |  |  |  |
| ICU mortality | 76 (9.0) | 17 (6.1) | 21 (7.5) | 38 (13.6) | 0.005 |
| Hospital mortality | 108 (12.9) | 24 (8.6) | 25 (8.9) | 59 (21.1) | < 0.001 |
| 28-day mortality | 132 (15.7) | 30 (10.7) | 33 (11.8) | 69 (24.6) | < 0.001 |
| 365-day mortality | 277 (33.0) | 68 (24.3) | 86 (30.7) | 123 (43.9) | < 0.001 |

Data are presented as mean ±SD, medians (interquartile ranges) or numbers (percentages)

SII, systemic immune-inflammation index; MBP, mean blood pressure; SPO_2_, percutaneous oxygen saturation; CHF, congestive heart failure; PVD, peripheral vascular disease; CVD, cerebrovascular disease; CPD, chronic pulmonary disease; SOFA, sequential organ failure assessment; BUN, blood urea nitrogen; WBC, white blood cell count; RRT, renal replacement therapy; ICU, intensive care unit.

Table S5 Characteristics and outcomes of participants categorized by SIRI

| Variables | Total  (n = 840) | SIRI | | | *P* -value |
| --- | --- | --- | --- | --- | --- |
|  |  | Tertile 1  (n = 280) | Tertile 2  (n = 280) | Tertile 3  (n = 280) |  |
| Gender, n (%) | |  |  |  | 0.366 |
| Female | 308 (36.7) | 110 (39.3) | 94 (33.6) | 104 (37.1) |  |
| Male | 532 (63.3) | 170 (60.7) | 186 (66.4) | 176 (62.9) |  |
| Age (years) | 71.2 ± 11.6 | 70.7 ± 11.3 | 71.3 ± 11.6 | 71.7 ± 11.9 | 0.606 |
| Race, n (%) | |  |  |  | 0.410 |
| White | 483 (57.5) | 152 (54.3) | 166 (59.3) | 165 (58.9) |  |
| Others | 357 (42.5) | 128 (45.7) | 114 (40.7) | 115 (41.1) |  |
| *Vital signs* |  |  |  |  |  |
| Heart rate (beats/min) | 81.7 ± 15.2 | 79.1 ± 12.5 | 80.7 ± 14.6 | 85.3 ± 17.4 | < 0.001 |
| Respiratory rate (breaths/min) | 19.2 ± 3.5 | 18.3 ± 3.1 | 19.2 ± 3.4 | 20.1 ± 3.7 | < 0.001 |
| MBP (mmHg) | 76.9 ± 10.7 | 75.8 ± 10.4 | 78.6 ± 11.9 | 76.3 ± 9.6 | 0.005 |
| SPO_2_ (%) | 97.1 ± 2.1 | 97.4 ± 2.0 | 97.2 ± 1.9 | 96.8 ± 2.3 | 0.003 |
| *Comorbidities, n (%)* |  |  |  |  |  |
| Myocardial infarction | 307 (36.5) | 106 (37.9) | 92 (32.9) | 109 (38.9) | 0.281 |
| CHF | 472 (56.2) | 133 (47.5) | 163 (58.2) | 176 (62.9) | < 0.001 |
| PVD | 130 (15.5) | 39 (13.9) | 40 (14.3) | 51 (18.2) | 0.298 |
| CVD | 120 (14.3) | 50 (17.9) | 36 (12.9) | 34 (12.1) | 0.109 |
| CPD | 211 (25.1) | 66 (23.6) | 62 (22.1) | 83 (29.6) | 0.094 |
| Malignant cancer | 87 (10.4) | 30 (10.7) | 21 (7.5) | 36 (12.9) | 0.112 |
| Severe liver disease | 24 (2.9) | 10 (3.6) | 6 (2.1) | 8 (2.9) | 0.598 |
| *Scoring systems* |  |  |  |  |  |
| Charlson comorbidity index | 9.5 ± 2.1 | 9.3 ± 2.1 | 9.4 ± 2.1 | 9.8 ± 2.2 | 0.005 |
| SOFA | 6.3 ± 3.3 | 6.4 ± 3.1 | 6.1 ± 3.3 | 6.6 ± 3.5 | 0.141 |
| *Laboratory parameters* |  |  |  |  |  |
| Potassium (mmol/L) | 4.6 ± 0.8 | 4.5 ± 0.8 | 4.6 ± 0.9 | 4.6 ± 0.8 | 0.641 |
| Sodium (mmol/L) | 138.2 ± 5.4 | 139.2 ± 4.1 | 138.4 ± 5.9 | 136.9 ± 5.8 | < 0.001 |
| Hemoglobin (g/dL) | 9.4 ± 2.0 | 8.9 ± 1.8 | 9.6 ± 2.1 | 9.7 ± 2.0 | < 0.001 |
| Bicarbonate (mmol/L) | 21.4 ± 4.8 | 22.0 ± 4.5 | 21.8 ± 4.4 | 20.4 ± 5.4 | < 0.001 |
| BUN (mg/dL) | 36.0 (23.0, 57.0) | 31.0 (19.0, 45.0) | 35.0 (24.0, 61.2) | 44.0 (29.0, 70.2) | < 0.001 |
| Creatinine (mmol/L) | 1.9 (1.3, 3.5) | 1.6 (1.1, 3.0) | 1.8 (1.3, 3.1) | 2.4 (1.5, 4.1) | < 0.001 |
| Glucose (mg/dL) | 149.5 (112.0, 201.8) | 126.0 (100.5, 159.0) | 156.0 (115.0, 205.5) | 178.5 (125.0, 247.5) | < 0.001 |
| WBC (10^9^/L) | 11.3 (8.2, 15.8) | 8.1 (6.1, 10.7) | 11.0 (8.7, 13.9) | 16.0 (12.3, 20.8) | < 0.001 |
| Blood culture positivity (%) | 57 (6.8) | 10 (3.6) | 10 (3.6) | 37 (13.2) | < 0.001 |
| *Treatment, n (%)* |  |  |  |  |  |
| Vasoactive agent | 382 (45.5) | 140 (50) | 118 (42.1) | 124 (44.3) | 0.155 |
| Antibiotic | 629 (74.9) | 215 (76.8) | 192 (68.6) | 222 (79.3) | 0.009 |
| RRT | 122 (14.5) | 32 (11.4) | 36 (12.9) | 54 (19.3) | 0.019 |
| *Death events, n (%)* |  |  |  |  |  |
| ICU mortality | 76 (9.0) | 11 (3.9) | 19 (6.8) | 46 (16.4) | < 0.001 |
| Hospital mortality | 108 (12.9) | 18 (6.4) | 25 (8.9) | 65 (23.2) | < 0.001 |
| 28-day mortality | 132 (15.7) | 22 (7.9) | 37 (13.2) | 73 (26.1) | < 0.001 |
| 365-day mortality | 277 (33.0) | 61 (21.8) | 86 (30.7) | 130 (46.4) | < 0.001 |

Data are presented as mean ±SD, medians (interquartile ranges) or numbers (percentages)

SIRI, systemic inflammation response index; MBP, mean blood pressure; SPO_2_, percutaneous oxygen saturation; CHF, congestive heart failure; PVD, peripheral vascular disease; CVD, cerebrovascular disease; CPD, chronic pulmonary disease; SOFA, sequential organ failure assessment; BUN, blood urea nitrogen; WBC, white blood cell count; RRT, renal replacement therapy; ICU, intensive care unit.

Table S6 Characteristics and outcomes of participants categorized by NLR

| Variables | Total  (n = 840) | NLR | | | *P* -value |
| --- | --- | --- | --- | --- | --- |
|  |  | Tertile 1  (n = 280) | Tertile 2  (n = 280) | Tertile 3  (n = 280) |  |
| Gender, n (%) | |  |  |  | 0.677 |
| Female | 308 (36.7) | 108 (38.6) | 102 (36.4) | 98 (35) |  |
| Male | 532 (63.3) | 172 (61.4) | 178 (63.6) | 182 (65) |  |
| Age (years) | 71.2 ± 11.6 | 71.4 ± 11.2 | 71.1 ± 12.0 | 71.1 ± 11.7 | 0.954 |
| Race, n (%) | |  |  |  | 0.636 |
| White | 483 (57.5) | 160 (57.1) | 156 (55.7) | 167 (59.6) |  |
| Others | 357 (42.5) | 120 (42.9) | 124 (44.3) | 113 (40.4) |  |
| *Vital signs* |  |  |  |  |  |
| Heart rate (beats/min) | 81.7 ± 15.2 | 79.2 ± 12.6 | 82.0 ± 14.9 | 83.9 ± 17.3 | < 0.001 |
| Respiratory rate (breaths/min) | 19.2 ± 3.5 | 18.5 ± 3.1 | 18.9 ± 3.3 | 20.2 ± 3.8 | < 0.001 |
| MBP (mmHg) | 76.9 ± 10.7 | 76.9 ± 10.8 | 77.1 ± 10.6 | 76.8 ± 10.8 | 0.959 |
| SPO_2_ (%) | 97.1 ± 2.1 | 97.4 ± 2.0 | 97.1 ± 2.0 | 96.8 ± 2.3 | 0.004 |
| *Comorbidities, n (%)* |  |  |  |  |  |
| Myocardial infarction | 307 (36.5) | 103 (36.8) | 94 (33.6) | 110 (39.3) | 0.371 |
| CHF | 472 (56.2) | 128 (45.7) | 161 (57.5) | 183 (65.4) | < 0.001 |
| PVD | 130 (15.5) | 34 (12.1) | 42 (15) | 54 (19.3) | 0.063 |
| CVD | 120 (14.3) | 51 (18.2) | 37 (13.2) | 32 (11.4) | 0.059 |
| CPD | 211 (25.1) | 56 (20) | 70 (25) | 85 (30.4) | 0.018 |
| Malignant cancer | 87 (10.4) | 31 (11.1) | 21 (7.5) | 35 (12.5) | 0.135 |
| Severe liver disease | 24 (2.9) | 8 (2.9) | 8 (2.9) | 8 (2.9) | 1.000 |
| *Scoring systems* |  |  |  |  |  |
| Charlson comorbidity index | 9.5 ± 2.1 | 9.1 ± 1.9 | 9.5 ± 2.1 | 9.8 ± 2.2 | < 0.001 |
| SOFA | 6.3 ± 3.3 | 6.2 ± 3.2 | 6.0 ± 3.2 | 6.8 ± 3.4 | 0.008 |
| *Laboratory parameters* |  |  |  |  |  |
| Potassium (mmol/L) | 4.6 ± 0.8 | 4.5 ± 0.8 | 4.6 ± 0.8 | 4.6 ± 0.9 | 0.331 |
| Sodium (mmol/L) | 138.2 ± 5.4 | 139.0 ± 4.7 | 138.6 ± 5.2 | 136.8 ± 5.9 | < 0.001 |
| Hemoglobin (g/dL) | 9.4 ± 2.0 | 9.2 ± 2.0 | 9.2 ± 2.1 | 9.7 ± 2.0 | 0.007 |
| Bicarbonate (mmol/L) | 21.4 ± 4.8 | 22.1 ± 4.0 | 21.4 ± 5.0 | 20.6 ± 5.3 | 0.001 |
| BUN (mg/dL) | 36.0 (23.0, 57.0) | 29.0 (19.0, 43.2) | 37.5 (25.0, 61.2) | 44.5 (29.0, 67.2) | < 0.001 |
| Creatinine (mmol/L) | 1.9 (1.3, 3.5) | 1.5 (1.1, 2.7) | 1.8 (1.3, 3.3) | 2.5 (1.5, 4.0) | < 0.001 |
| Glucose (mg/dL) | 149.5 (112.0, 201.8) | 130.0 (104.0, 161.8) | 153.0 (112.0, 212.8) | 177.5 (123.8, 244.0) | < 0.001 |
| WBC (10^9^/L) | 11.3 (8.2, 15.8) | 9.0 (6.5, 11.9) | 11.3 (8.8, 14.8) | 14.2 (10.9, 19.5) | < 0.001 |
| Blood culture positivity (%) | 57 (6.8) | 6 (2.1) | 12 (4.3) | 39 (13.9) | < 0.001 |
| *Treatment, n (%)* |  |  |  |  |  |
| Vasoactive agent | 382 (45.5) | 140 (50) | 118 (42.1) | 124 (44.3) | 0.155 |
| Antibiotic | 629 (74.9) | 201 (71.8) | 195 (69.6) | 233 (83.2) | < 0.001 |
| RRT | 122 (14.5) | 30 (10.7) | 36 (12.9) | 56 (20) | 0.005 |
| *Death events, n (%)* |  |  |  |  |  |
| ICU mortality | 76 (9.0) | 10 (3.6) | 25 (8.9) | 41 (14.6) | < 0.001 |
| Hospital mortality | 108 (12.9) | 16 (5.7) | 31 (11.1) | 61 (21.8) | < 0.001 |
| 28-day mortality | 132 (15.7) | 18 (6.4) | 40 (14.3) | 74 (26.4) | < 0.001 |
| 365-day mortality | 277 (33.0) | 63 (22.5) | 88 (31.4) | 126 (45) | < 0.001 |

Data are presented as mean ±SD, medians (interquartile ranges) or numbers (percentages)

NLR, neutrophil-to-lymphocyte ratio; MBP, mean blood pressure; SPO_2_, percutaneous oxygen saturation; CHF, congestive heart failure; PVD, peripheral vascular disease; CVD, cerebrovascular disease; CPD, chronic pulmonary disease; SOFA, sequential organ failure assessment; BUN, blood urea nitrogen; WBC, white blood cell count; RRT, renal replacement therapy; ICU, intensive care unit.

Table S7 Cox proportional hazard models for 365-day mortality

|  | Crude model | | Model 1 | | Model 2 | |
| --- | --- | --- | --- | --- | --- | --- |
| Variable | HR (95%CI) | *P* value | HR (95%CI) | *P* value | HR (95%CI) | *P* value |
| Continuous ln-SII | 1.35 (1.21-1.51) | <0.001 | 1.36 (1.22-1.52) | <0.001 | 1.34 (1.18-1.53) | <0.001 |
| Categories (SII tertile) | | | | | | |
| Tertile 1 | 1 (Ref) | | 1 (Ref) | | 1 (Ref) | |
| Tertile 2 | 1.31 (0.95-1.79) | 0.100 | 1.36 (0.99-1.87) | 0.059 | 1.53 (1.09-2.15) | 0.014 |
| Tertile 3 | 2.14 (1.59-2.88) | <0.001 | 2.22 (1.65-2.99) | <0.001 | 2.07 (1.46-2.93) | <0.001 |
| *P* for trend |  | <0.001 |  | <0.001 |  | <0.001 |
| Continuous ln-SIRI | 1.36 (1.24-1.49) | <0.001 | 1.35 (1.23-1.48) | <0.001 | 1.35 (1.19-1.53) | <0.001 |
| Categories (SIRI tertile) | | | | | | |
| Tertile 1 | 1 (Ref) | | 1 (Ref) | | 1 (Ref) | |
| Tertile 2 | 1.50 (1.08-2.08) | 0.015 | 1.52 (1.09-2.11) | 0.013 | 1.64 (1.15-2.34) | 0.006 |
| Tertile 3 | 2.62 (1.93-3.56) | <0.001 | 2.64 (1.95-3.59) | <0.001 | 2.69 (1.84-3.94) | <0.001 |
| *P* for trend |  | <0.001 |  | <0.001 |  | <0.001 |
| Continuous ln-NLR | 1.56 (1.37-1.77) | <0.001 | 1.58 (1.39-1.79) | <0.001 | 1.39 (1.20-1.61) | <0.001 |
| Categories (NLR tertile) | | | | | | |
| Tertile 1 | 1 (Ref) | | 1 (Ref) | | 1 (Ref) | |
| Tertile 2 | 1.50 (1.09-2.08) | 0.014 | 1.53 (1.11-2.11) | 0.010 | 1.43 (1.01-2.02) | 0.045 |
| Tertile 3 | 2.46 (1.82-3.33) | <0.001 | 2.60 (1.92-3.52) | <0.001 | 2.04 (1.43-2.91) | <0.001 |
| *P* for trend | <0.001 | | <0.001 | | <0.001 | |

In sensitivity analysis, inﬂammatory indicators were converted from a continuous variable to a categorical variable (tertiles)

Crude model: no covariates was adjusted

Model 1: adjusted for age, gender, race

Model 2: adjusted for age, gender, race, heart rate, respiratory rate, mean blood pressure, percutaneous oxygen saturation, myocardial infarction, congestive heart failure, peripheral vascular disease, cerebrovascular disease, chronic pulmonary disease, malignant cancer, severe liver disease, Charlson comorbidity index, sequential organ failure assessment, potassium, sodium, hemoglobin, bicarbonate, blood urea nitrogen, creatinine, glucose, white blood cell count, blood culture, vasoactive agent, antibiotic, renal replacement therapy

Table S8 Sensitivity analyses were performed after multiple imputation of missing data.

| Variable | Crude model | | Model 2 | |
| --- | --- | --- | --- | --- |
|  | HR (95%CI) | *P* value | HR (95%CI) | *P* value |
| Ln-SII | 1.43 (1.22-1.68) | <0.001 | 1.39 (1.16-1.67) | 0.001 |
| Ln-SIRI | 1.48 (1.30-1.70) | <0.001 | 1.36 (1.14-1.63) | 0.001 |
| Ln-NLR | 1.74 (1.46-2.09) | <0.001 | 1.48 (1.20-1.84) | <0.001 |

Crude model: no covariates was adjusted.

Model 2: adjusted for age, gender, race, heart rate, respiratory rate, mean blood pressure, percutaneous oxygen saturation, myocardial infarction, congestive heart failure, peripheral vascular disease, cerebrovascular disease, chronic pulmonary disease, malignant cancer, severe liver disease, Charlson comorbidity index, sequential organ failure assessment, potassium, sodium, hemoglobin, bicarbonate, blood urea nitrogen, creatinine, glucose, white blood cell count, blood culture, vasoactive agent, antibiotic, renal replacement therapy.

SII, systemic immune-inflammation index; SIRI, systemic inflammation response index; NLR, neutrophil-to-lymphocyte ratio

Table S9 Harrell’s C-index of SII, SIRI and NLR

|  | Harrell’s C-index (95%CI) |
| --- | --- |
| SII | 0.626 (0.575, 0.676) |
| SIRI | 0.665 (0.618, 0.712) |
| NLR | 0.668 (0.624, 0.713) |

SII, systemic immune-inflammation index; SIRI, systemic inflammation response index; NLR, neutrophil-to-lymphocyte ratio.

Table S10 NRI index of SII, SIRI and NLR

| Comparison | Continuous NRI Estimate (95%CI) | *P*-value |
| --- | --- | --- |
| SIRI vs. SII | 0.136 (0.060, 0.255) | 0.012 |
| SII vs. NLR | 0.104 (-0.186, 0.272) | 0.623 |
| SIRI vs. NLR | 0.202 (0.103, 0.303) | 0.004 |

NRI, net reclassification index; SII, systemic immune-inflammation index; SIRI, systemic inflammation response index; NLR, neutrophil-to-lymphocyte ratio.

## Supplementary Figures

Figure S1. Plot of Schoenfeld residuals against time in the Cox regression model. (A), (B), (C) Plot of Schoenfeld residuals for SII, SIRI and NLR on 28-day mortality, respectively; (D), (E), (F) Plot of Schoenfeld residuals for SII, SIRI and NLR on 365-day mortality, respectively. SII, systemic immune-inflammation index; SIRI, systemic inflammation response index; NLR, neutrophil-to-lymphocyte ratio.

Figure S2. Forest plots of hazard ratios of systemic immune-inflammation index for the 28-day mortality in different subgroups. HR, hazard ratio; CI, confidence interval.

Figure S3. Forest plots of hazard ratios of systemic inflammation response index for the 28-day mortality in different subgroups. HR, hazard ratio; CI, confidence interval.

Figure S4. Forest plots of hazard ratios of neutrophil-to-lymphocyte ratio for the 28-day mortality in different subgroups. HR, hazard ratio; CI, confidence interval.

Figure S5. The time-dependent receiver operating characteristic of different systemic inflammatory indicators with the mortality of the diabetic kidney disease population in intensive care unit. (A) systemic immune-inflammation index; (B) systemic inflammation response index; (C) neutrophil-to-lymphocyte ratio.

Figure S6. Calibration plots (A) SII, (B) SIRI, (C) NLR. SII, systemic immune-inflammation index; SIRI, systemic inflammation response index; NLR, neutrophil-to-lymphocyte ratio.
