# Supplementary figures and images for: Association between systemic inflammatory indicators on admission and mortality in critically ill patients with diabetic kidney disease based on the MIMIC-IV database: a cohort study
Source: Front Endocrinol (Lausanne). 2025 May 30;16:1503667. doi: 10.3389/fendo.2025.1503667 (PMC12162917; doi:10.3389/fendo.2025.1503667)

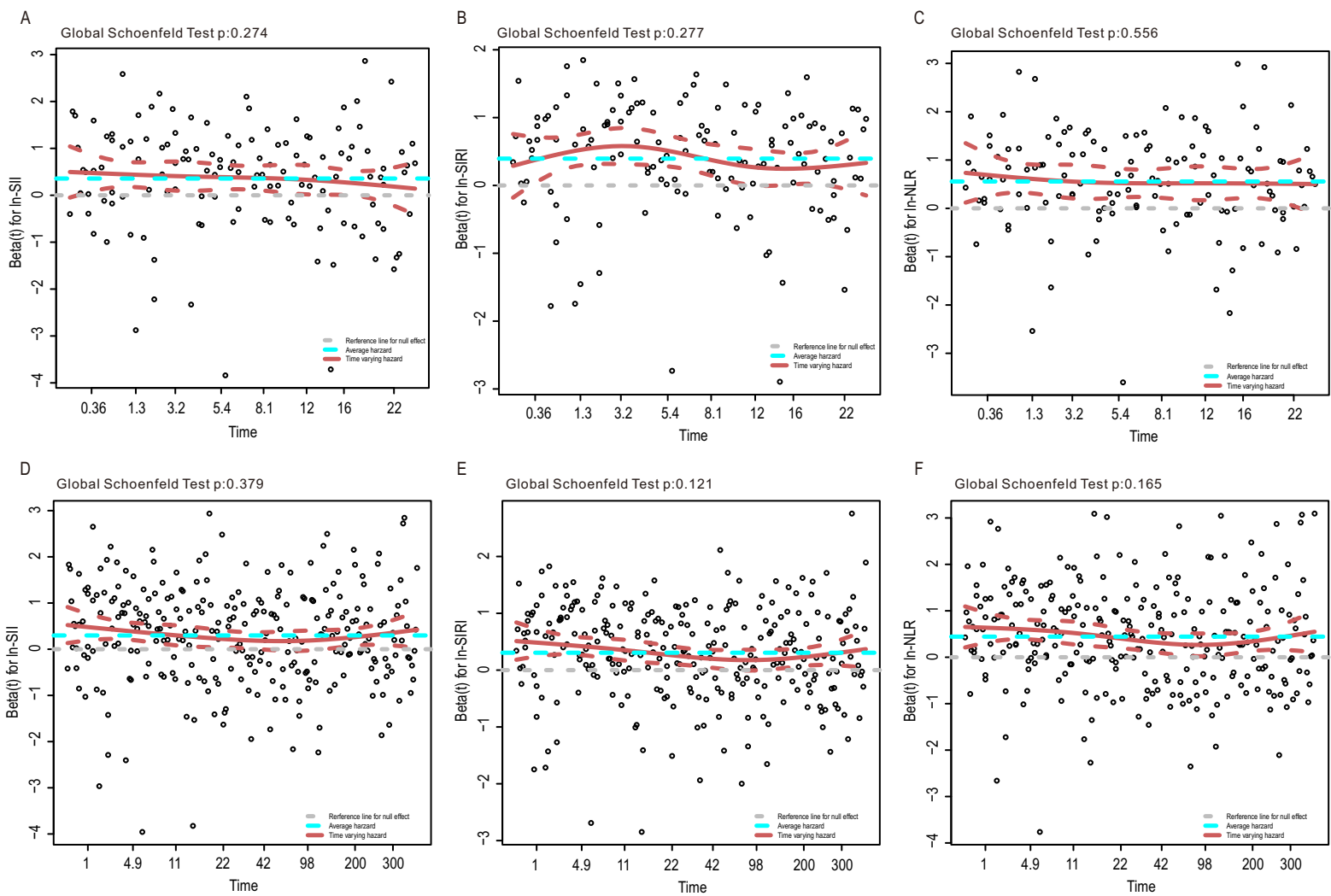

Supplement: Supplementary file 2 [file Image1.pdf]

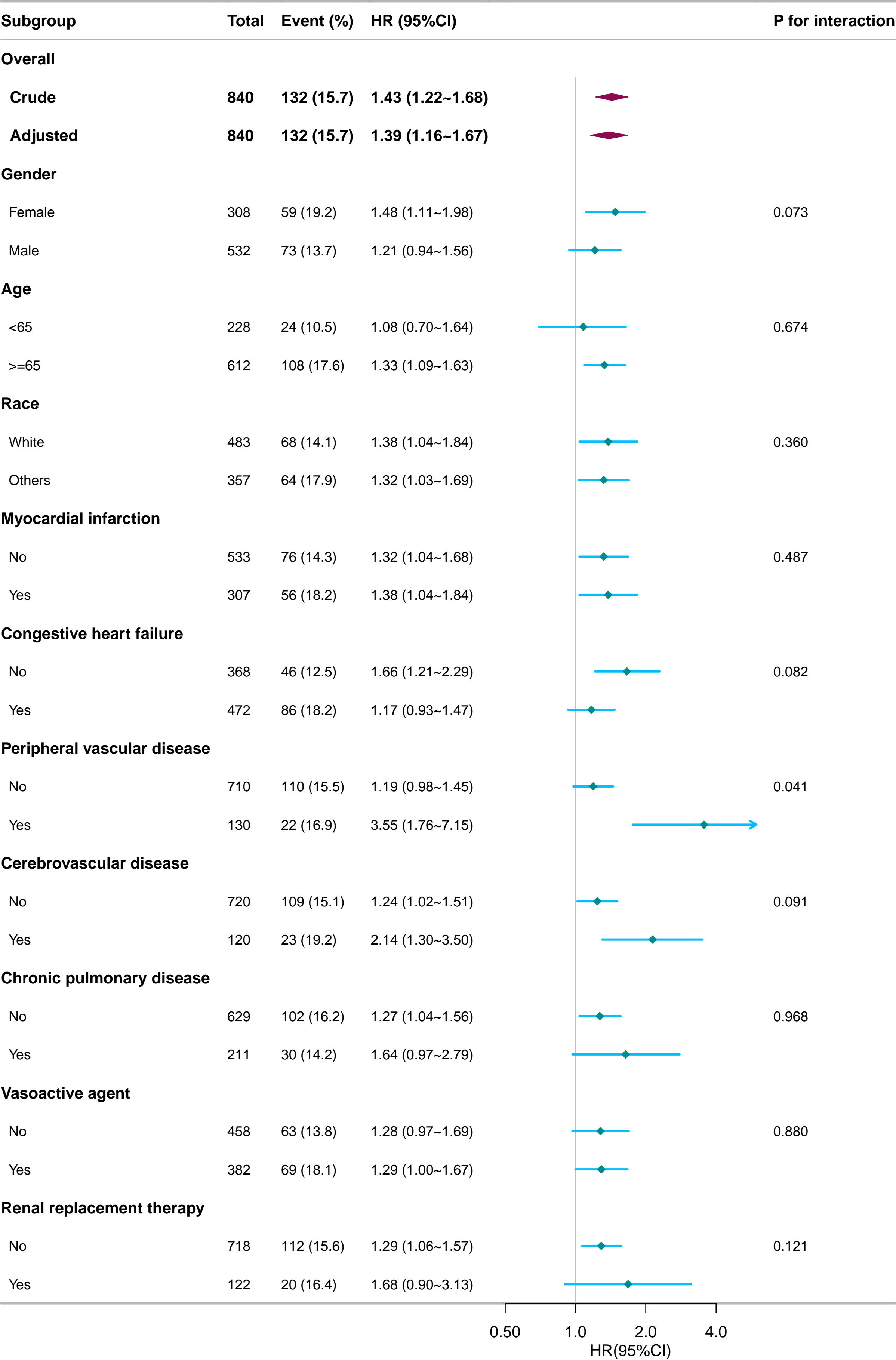

Supplement: Supplementary file 3 [file Image2.pdf]

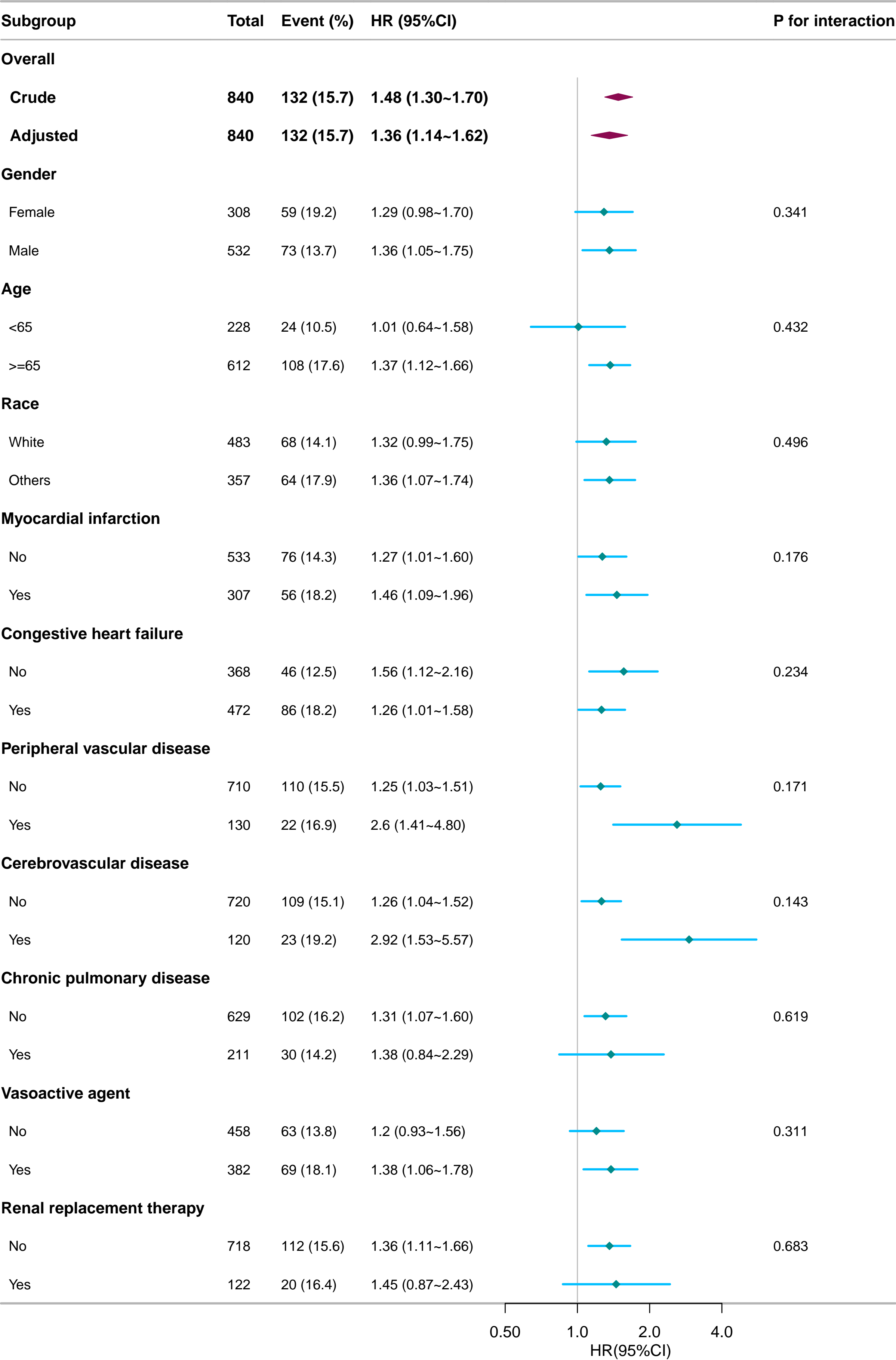

Supplement: Supplementary file 4 [file Image3.pdf]

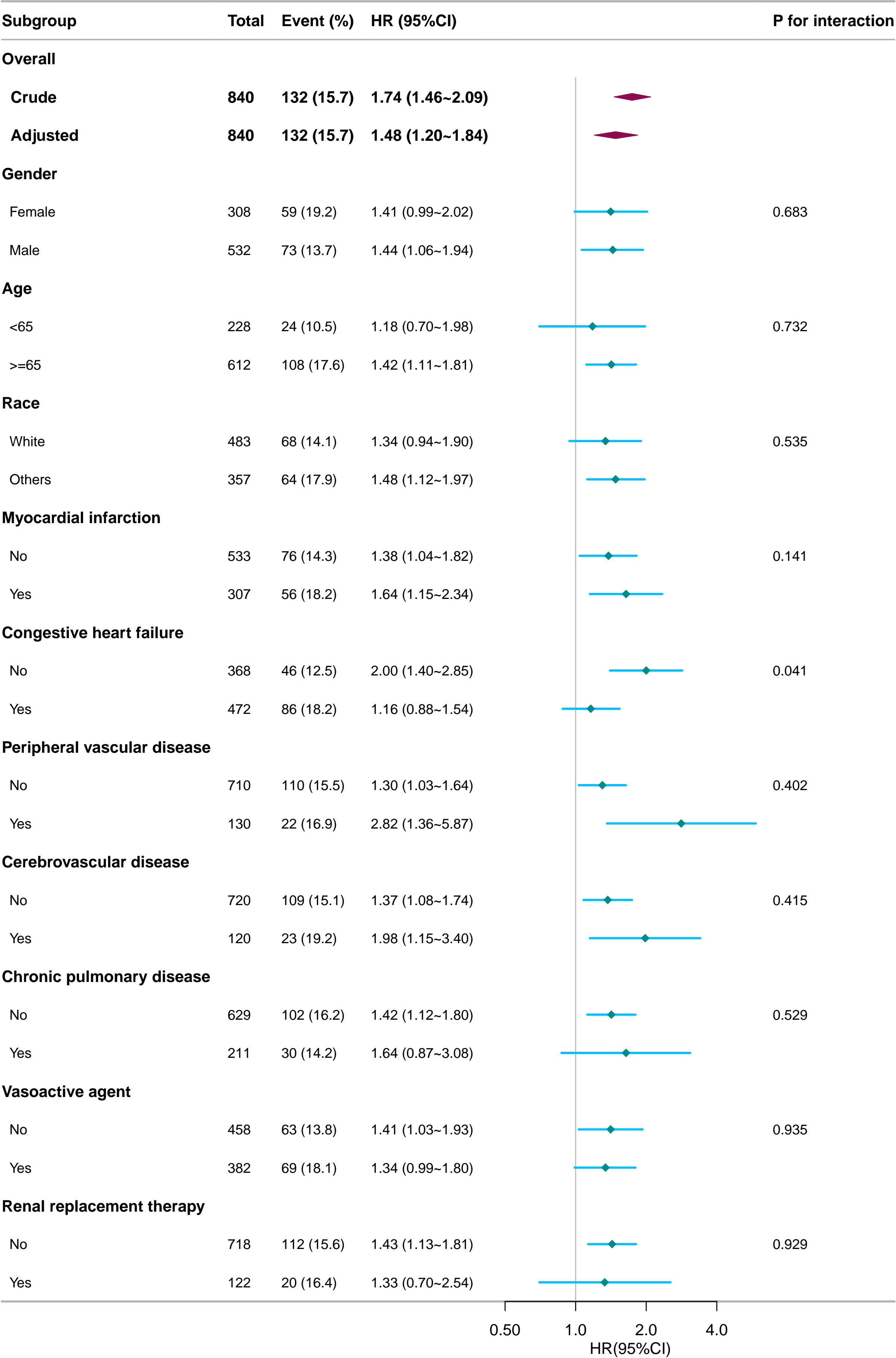

Supplement: Supplementary file 5 [file Image4.pdf]

A

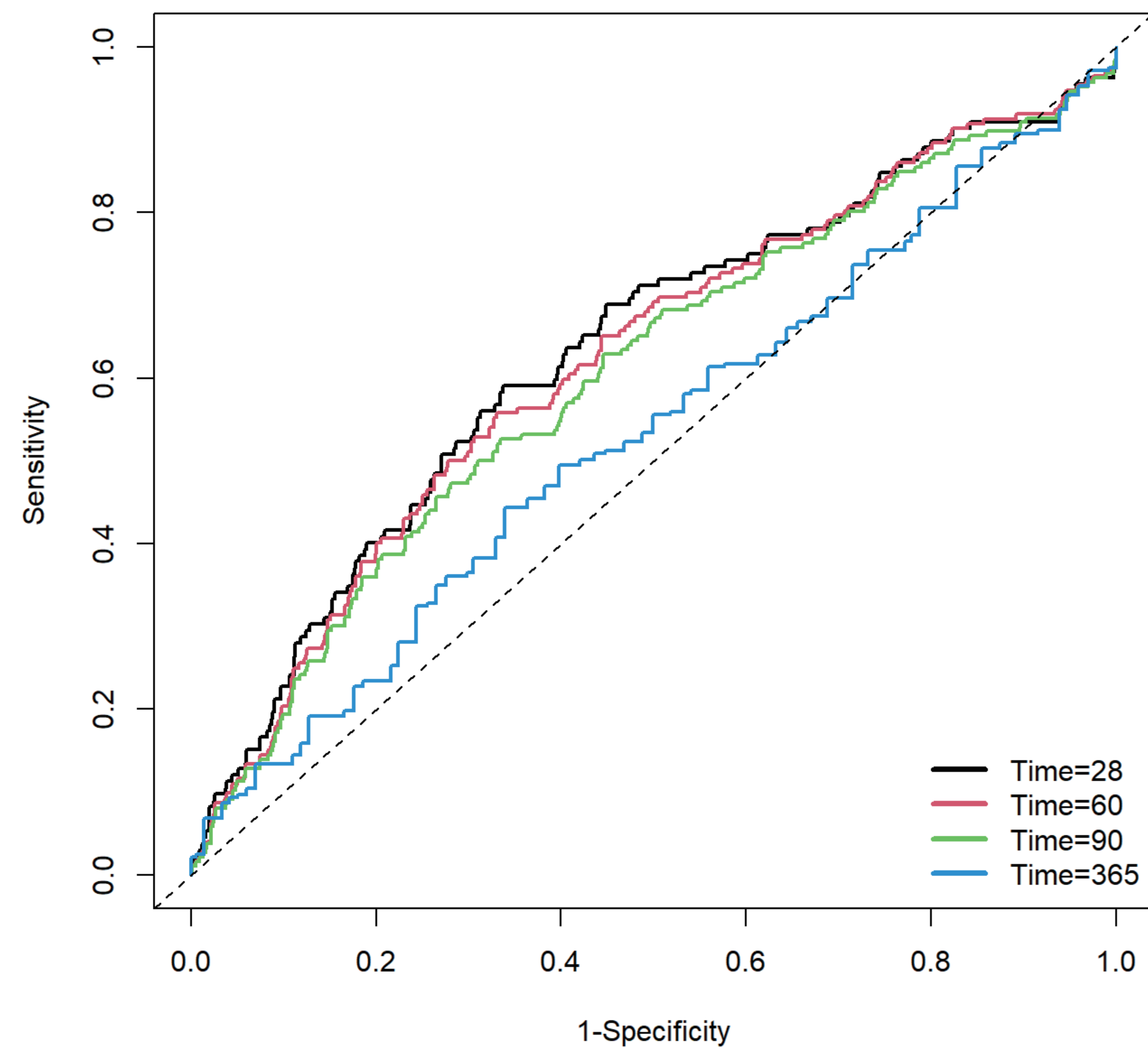

B

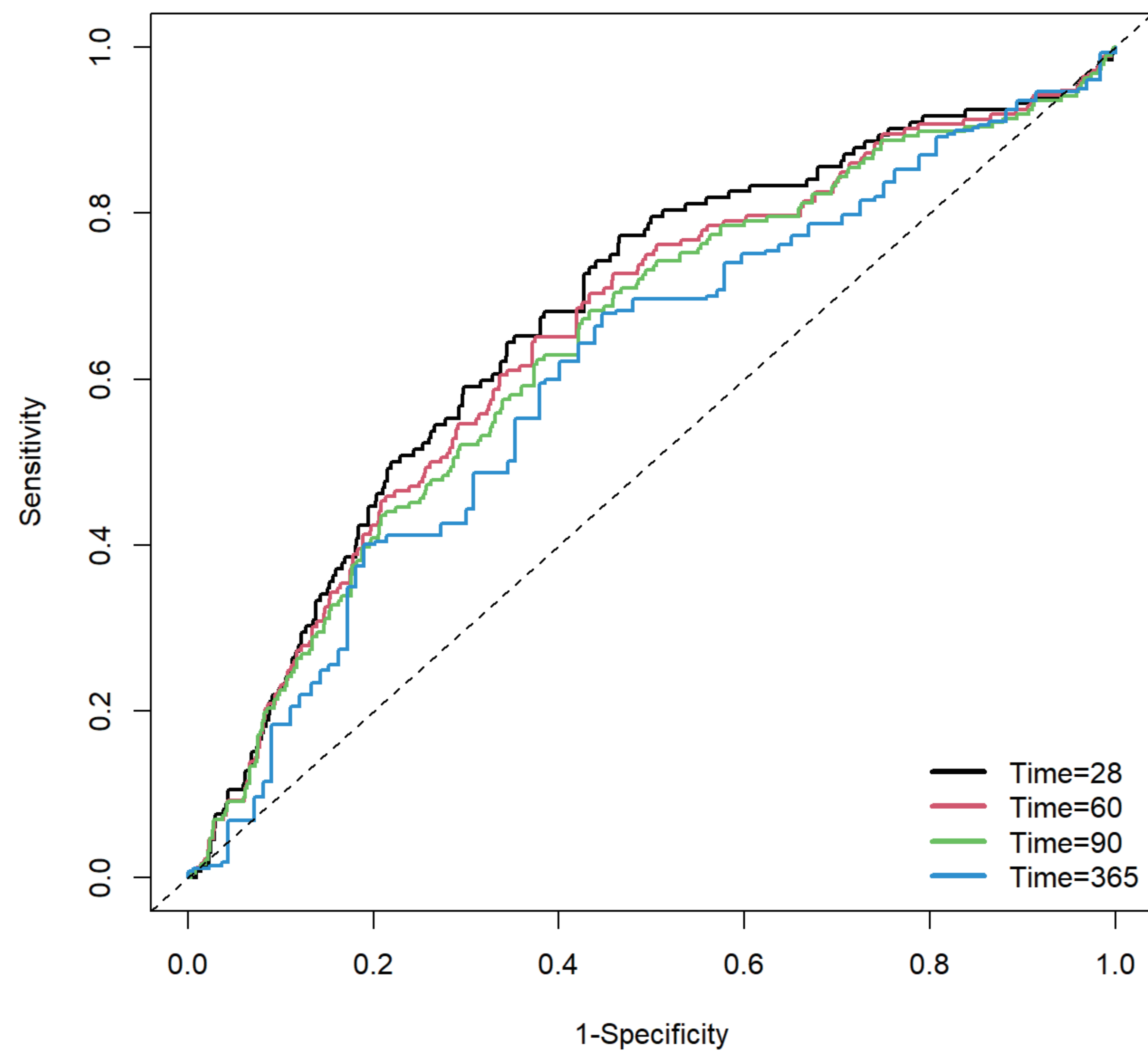

C

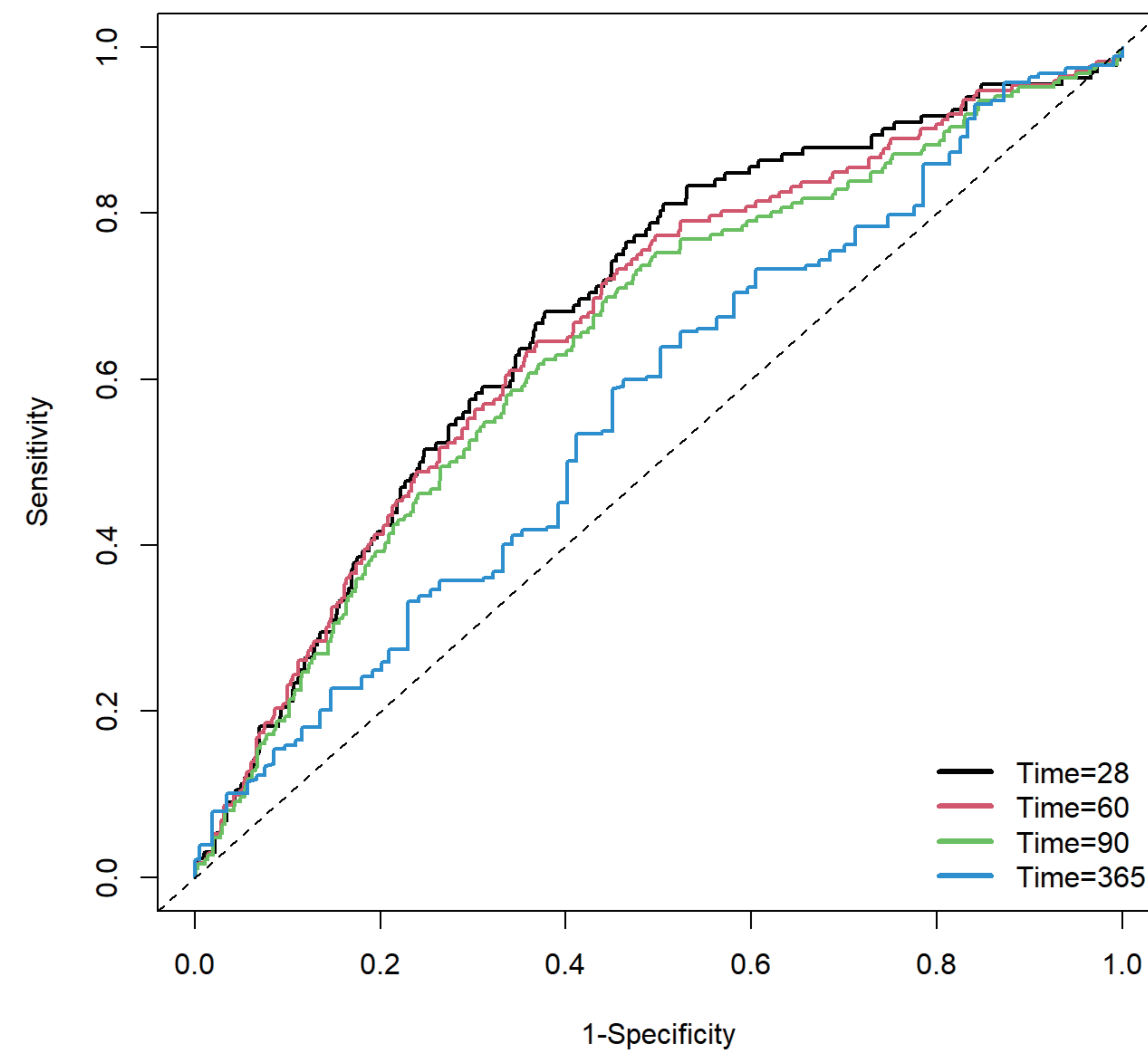

Supplement: Supplementary file 6 [file Image5.pdf]

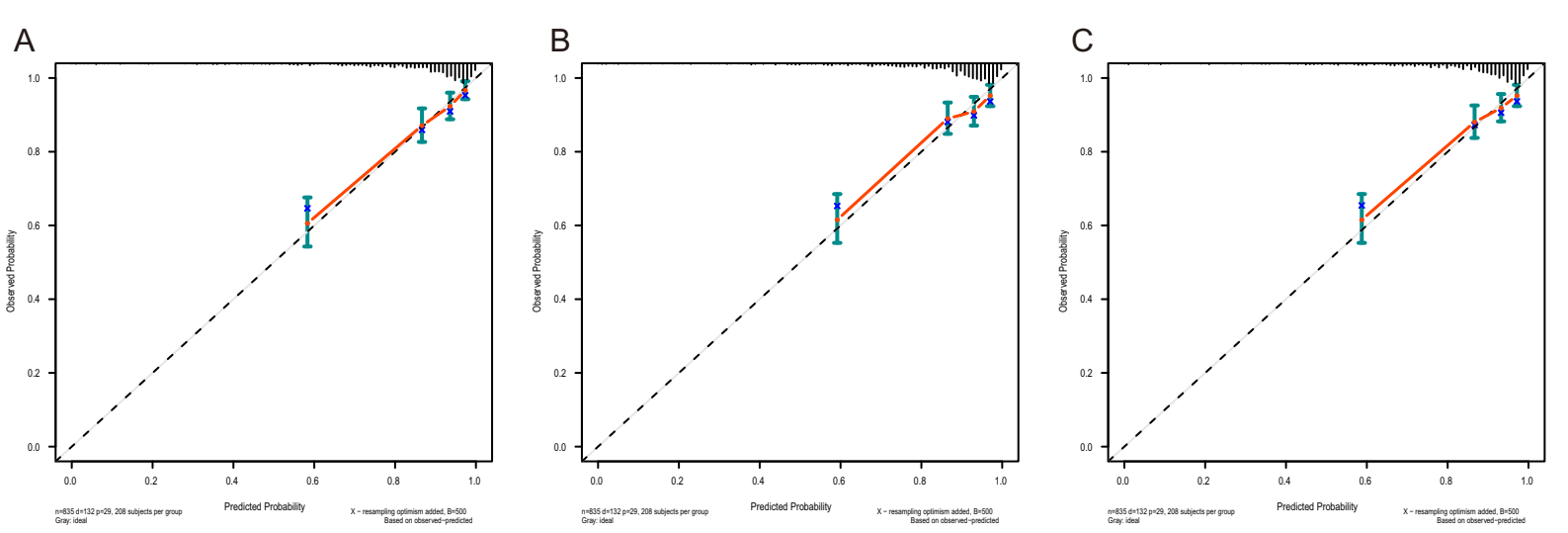

Supplement: Supplementary file 7 [file Image6.pdf]
